# Associations between dairy fat intake, milk-derived free fatty acids, and cardiometabolic risk in Dutch adults

**DOI:** 10.1007/s00394-022-02974-0

**Published:** 2022-08-05

**Authors:** Katherine J. Li, Elske M. Brouwer-Brolsma, Charlotte Fleuti, René Badertscher, Guy Vergères, Edith J. M. Feskens, Kathryn J. Burton-Pimentel

**Affiliations:** 1 Division of Human Nutrition and Health, Department of Agrotechnology and Food Science, Wageningen University & Research, Wageningen, The Netherlands; 2 Agroscope, Federal Department of Economic Affairs, Education and Research (EAER), Federal Office for Agriculture (FOAG), Bern, Switzerland

**Keywords:** Dairy, Free fatty acids, Biomarker, Cardiometabolic disease risk

## Abstract

**Purpose:**

Milk-derived free fatty acids (FFAs) may act as both biomarkers of intake and metabolic effect. In this study we explored associations between different types of dairy consumption, a selection of milk-derived free fatty acids, and cardiometabolic disease (CMD) risk factors.

**Methods:**

Sixty-seven FFAs were quantified in the plasma of 131 free-living Dutch adults (median 60 years) using gas chromatography-flame ionization detector. Intakes of different dairy foods and groups were assessed using a food frequency questionnaire. Twelve different CMD risk factors were analyzed. Multiple linear regressions were used to evaluate the associations under study.

**Results:**

Based on the fully adjusted models, 5 long-chain unsaturated FFAs (C18:1 t13 + c6 + c7 + u, C18:2 c9t11 + u, C20:1 c11, C20:3 c8c11c14, and C20:4 c5c8c11c14), 2 medium-chain saturated FFAs (C15, C15 *iso*), and a *trans* FFA (C16:1 t9) were positively associated with at least one variable of dairy intake, as well as plasma total and LDL cholesterol, blood pressure, and SCORE (*p* ≤ 0.05). A long-chain PUFA associated with high-fat fermented dairy intake (C18:2 t9t12), was negatively associated with serum triglyceride levels, and a long-chain saturated FFA associated with cheese intake (C18:1 u1) was negatively associated with plasma LDL cholesterol and serum triglyceride levels. No clear associations were observed between dairy intake and CMD risk factors.

**Conclusion:**

Milk-derived FFAs could act as sensitive biomarkers for dairy intake and metabolism, allowing the association between dairy and CMD risk to be more precisely evaluated.

**Supplementary Information:**

The online version contains supplementary material available at 10.1007/s00394-022-02974-0.

## Introduction

Cardiometabolic diseases (CMDs), encompassing cardiovascular disease and type II diabetes, represent one of the largest health and socioeconomic burdens to modern society. In Europe, cardiovascular disease morbidity affects more than 85 million people, leading to  ~ 4 million annual deaths [1]. Proper nutrition is considered the primary lifestyle approach for preventing and managing CMD risk. Diets abundant in fruits, vegetables, whole grains, nuts, and legumes contribute to lowering CMD risk, while consumption of processed meats, refined grains, and sugar-sweetened beverages are considered detrimental [2]. Particular nutrients in foods have also been adversely associated with CMD risk, including sugar, sodium, *trans* (unsaturated) fat, and saturated fat [2].

Dairy products have had a contested role in the dietary management of CMDs due to suggested beneficial as well as adverse health effects. While dairy foods are rich in macro- and micronutrients considered important for growth and development, they can also have a high saturated fat content (contributing 25–30% of all saturated fat intake in the European diet) [3]. Earlier studies conducted in the 1960–70 s have reported that saturated fat adversely affects low-density lipoprotein cholesterol (LDL-C) levels in blood, which in turn increases CMD pathogenesis and progression [3, 4]. In view of this, many subsequent observational studies have compared high- and low-fat dairy intake on CMD risk, but the findings have been equivocal. A meta-analysis of prospective cohort studies revealed that total and low-fat dairy intake (but not high-fat dairy intake) was associated with a lower risk of hypertension [5], while several studies have revealed the merits of consuming full-fat dairy products on reducing central adiposity risk [6, 7] and increasing serum high-density lipoprotein cholesterol (HDL-C) [8]. In a recent systematic review of randomized controlled trials, Duarte et al. [9] reported that the consumption of dairy products (as a source of saturated fat) may improve some CMD risk factors compared with consumption of other animal sources of saturated fat. This finding lends support to the importance of considering the whole dairy matrix on disease outcomes. An increasing body of evidence also supports the differential role of distinct dairy foods with unique nutritional and/or microbial profiles on CMD risk [10]. For instance, consumption of yoghurt, a fermented dairy food, has been consistently associated with a reduced risk of type II diabetes [11] and combined cardiovascular disease [12].

A limitation of the above studies (which could partly explain the equivocal findings) is the reliance on subjective, self-reported dietary assessment methods. Here, food intake biomarkers (FIBs) could act as a more objective and accurate strategy for estimating dairy intake, and can lead to more consistent findings for associations between dairy intake and CMD risk factors [13]. Several fatty acids have been proposed as FIBs for dairy fat intake, including pentadecanoic acid (C15), heptadecanoic acid (C17), and *trans-*palmitoleic acid (C16:1 t9) [14–20], but these FIBs have not been thoroughly validated, and/or cannot discriminate the intake of specific dairy foods. Additionally, milk fat is a highly complex mixture of several thousand species of lipids, including  ~ 400 fatty acids, which have distinct physiological importance and nutritive potential [21, 22]. Longer chain saturated fatty acids (e.g., C14, C16) as well as certain *trans* fatty acids are known to positively associate with total and LDL-C [23] and type II diabetes risk [24], while saturated fatty acids with shorter chain length (e.g., C6-C10), monounsaturated, and polyunsaturated fatty acids (MUFA and PUFA) are generally regarded as cardio-protective [25–27]. Surprisingly little is known about the relationships between milk-derived fatty acids with dairy intake, or how they associate with CMD risk. Recently, Drouin-Cartier et al. [25] evaluated plasma metabolite profiles associated with total dairy intake and risk of type II diabetes, and found 38 metabolites associated with dairy intake and lower type II diabetes risk. However, such comprehensive studies are fairly scarce and there remains a need to consider the interplay between different dairy foods, lipids and metabolites, and CMD risk factors.

The objective of the current study is therefore to evaluate associations between dairy intake, milk-derived free fatty acids (FFAs), and CMD risk parameters in a free-living adult population. To achieve a comprehensive analysis, we targeted 67 FFAs with high abundance in milk and looked at their associations with different dairy intake groups (dairy fat, fermented and non-fermented dairy, and specific dairy foods), and a wide range of CMD risk factors and composite risk scores.

## Methods

### Study design and population

The Nutrition Questionnaires plus (NQplus) study is a prospective cohort study comprising 2048 Dutch adults (20–70 years) living in or around Wageningen, The Netherlands. NQplus was initiated as an ‘add-on’ study to the National Dietary Assessment Reference Database (NDARD) project, and full details of NQplus and NDARD have been published previously [28, 29]. Participants were recruited and enrolled in the study between June 2011 and February 2013. Extensive data were collected at baseline, including an assessment of habitual dietary intake by food frequency questionnaire (FFQ) and/or 24-h recall, background demographics, health, anthropometric, and lifestyle data. Fasting blood samples and 24-h urine samples were also collected. The study was approved by the ethical committee of Wageningen University & Research and conducted in agreement with the Declaration of Helsinki. Written informed consent was obtained from all participants prior to the start of the study.

For the present study, we selected *n* = 131 NQplus participants for targeted FFA analysis from *n* = 228 who had a plasma sample collected within ± 14 days of completing an FFQ [30]. The selection of this smaller group was necessary to attain a number of samples that could be reasonably measured within time and economic restraints. To ensure a balance of participants across all dairy groups/foods assessed,  ~ 20 participants were randomly selected from the low (Q1), mid (Q3), and high (Q5) quintiles of intake of each dairy group/food assessed.

### Food frequency questionnaire and levels of dairy consumption

A full description of the semi-quantitative FFQ used to assess habitual dietary intake has been described in the study design papers for NQplus and NDARD [28, 29]. The semi-quantitative FFQ was self-administered and completed online, with ten frequency categories ranging from ‘never’ to ‘6–7 days per week’. Portion sizes were estimated using commonly used household measures. Total food or nutrient intakes (in g/day) were determined by multiplying consumption frequency by portion size and nutrient content as defined in the Dutch food composition tables [31]. Out of 216 total food items in the FFQ, 39 were identified as dairy products, which were further classified into dairy subgroups (Table S1). This FFQ has been previously validated for various dairy foods and food groups, including milk, yogurt, cheese, total fermented dairy (FD), and total non-fermented dairy (NFD) (against multiple 24-h recalls) [32], which are used in the current study for evaluation of the respective candidate FIBs. Total dairy, FD, and NFD groups were further stratified into high-fat groups, which included all full-fat dairy products, and low-fat groups, which included semi-skim and skim dairy products. Fat content (g/100 g) for all dairy products was determined based on the values reported in the Dutch Food Composition Table [31] and classifications of products as skim, semi-skim, and full-fat were based on the guidelines set by the Dutch Dairy Commodities Act (see Table S1).

### Cardiometabolic disease risk parameters

A full description of the CMD risk parameters collected for NQplus has been described previously [29]. Height and weight were determined using a stadiometer (SECA, Germany, nearest 0.1 cm) and a digital weighing scale (SECA, nearest 0.1 kg), respectively. BMI was calculated by dividing weight (in kg) by height (in m^2^). Waist circumference was determined using a non-flexible measuring tape (SECA 201, nearest 0.5 cm); measurements were taken twice and averaged. Systolic and diastolic blood pressure (SBP and DBP) was measured using a digital blood pressure monitor (IntelliSense HEM-907, Omron Healthcare, USA); the first measurement was omitted and the second to up to the sixth measurement were averaged. Fasting plasma glucose, total, HDL-C, and serum triglycerides were analyzed using Dimension Vista 1500 automated analyser (Siemens, Erlangen, Germany) or Roche Modular P800 chemistry analyser (Roche Diagnostics, Indianapolis, USA). Plasma LDL-C was calculated with the Friedewald equation [33]. Blood hemoglobin A1c (HbA1c) was determined with HPLC (ADAMS A1c HA-8160 analyser, A. Menarini Diagnostics). Participants were characterized as having hypertension, suboptimal cholesterol, or type II diabetes based on the cut-offs and definitions described in relevant guidelines of the European Society of Cardiology/European Atherosclerosis Society (ESC/EAS) [34–36], and having metabolic syndrome based on the harmonized guidelines of the International Diabetes Federation (IDF) et al. [37].

Two further composite risk scores were included in our analyses. Firstly, a continuous metabolic syndrome (MetS) score was constructed based on summed age- and sex-adjusted standardized residuals (*z*-scores) of all individual MetS parameters that incorporates plasma glucose, SBP, DBP, HDL-C, serum triglycerides, waist circumference as risk factors [38–40]. Since HDL-C is inversely associated with CMD risk, residuals for this parameter were multiplied by − 1 prior to summing. Secondly, the 10-year risk of fatal cardiovascular disease was evaluated using the European Systematic COronary Risk Evaluation (SCORE) low-risk country chart [41, 42], which incorporates sex, age, smoking status, SBP, and total cholesterol as risk factors. For the calculation of SCORE, a ‘smoker’ was considered to encompass current smokers and former smokers who quit  > 35 years old, and ‘non-smoker’ as never smokers and former smokers who quit  < 35 years old, as this age cut-off has been previously documented as a critical smoking cessation age to prolong life expectancy [43].

### Covariates

Data on education level, smoking status, physical activity, and alcohol consumption were collected via standardized questionnaires [29]. Participants were classified as having ‘low’, ‘intermediate’, or ‘high’ education based on their highest levels of completed education (no education or primary/lower vocational education, lower secondary or intermediate vocational education, or higher secondary/vocational education or university). The criteria used to define ‘smoker’ and ‘non-smoker’ are outlined above. Information on the participants’ usual physical activity (min/week spent on sedentary, light, moderate and vigorous intensity activities) over the past 4 weeks was obtained using the validated Activity Questionnaire for Adults and Adolescents (AQuAA) [44]. Intake levels of alcohol and different foods were assessed by FFQ. Covariate selection was based on the current scientific literature and statistical testing (as described in the statistical analysis).

### Measurement of free fatty acid concentrations in plasma

Ethylenediaminetetraacetic acid (EDTA) plasma collected for NQplus was stored in the biobank at − 80 °C. The targeted panel comprised 67 FFAs previously detected with the highest abundance in the milk fat of Swiss cows [45]. In addition, several FFA groups were determined based on the sums of individual FFAs (Table S2). Immediately prior to analysis, plasma samples were thawed on ice and were prepared for analysis by adding 15 µL of the internal standard (C13, 7 µg/15 µL) to 100 µL of plasma, followed by methylation of FFAs with MeOH/HCl at 25 °C for 45 min using methods described previously [46, 47]. A post-reaction treatment for neutralization was performed with 350 µL Na_2_CO_3_, and extraction was performed with 300 µL hexane. The FFA concentrations in plasma were determined using 0.5 µL injection of the hexane solution and analyzed using an Agilent 6890 high-resolution gas chromatograph equipped with a capillary column (100-m CP-Sil 88, Varian BV, Middleburg, Netherlands) and a flame ionization detector.

### Statistical analysis

Participant characteristics are reported as mean (standard deviation) for normally distributed variables, or medians (interquartile range) for skewed variables. To permit comparability with a previous study where we assessed correlations between plasma C15 and C17 with various dairy groups [30], Spearman’s correlation coefficients (*r*_s_) were calculated as an initial step to assess the strength of the associations between dairy intakes (energy-adjusted g/day) and FFA concentrations (mg/L plasma); *p*-values are presented as raw and false discovery rate (FDR)-adjusted using the method of Benjamini and Hochberg [48]. Correlation coefficients of  ≥ 0.50 were considered good, 0.20–0.49 as moderate, and  < 0.20 as poor (39).

Multivariable adjusted linear regression and restricted cubic spline regression were used to evaluate the associations between self-reported dairy intakes, FFAs, and CMD risk factors. The assumption of linear relationships between exposure and outcome variables were tested using likelihood ratio tests of model deviance and Wald tests of spline coefficients. If tests were statistically significant, associations were visually inspected to confirm the presence of a true non-linear relationship and not due to artificial curves driven by outliers. All tests for non-linearity revealed that the dose–response association of dairy intake with FFAs and CMD risk parameters, and between FFAs and CMD risk parameters, could be considered linear. Thus, only linear regressions are presented in the results. Prior to association analyses, intakes of dairy foods were energy-adjusted using the commonly used residual method [49]. FFAs that were not detected in at least a third of participants were removed to select the most suitable candidate FIBs of dairy intake. CMD risk parameters acting as dependent variables that were not normally distributed (BMI, HbA1c, plasma glucose, serum triglycerides, and SCORE) were log transformed. Additionally, all variables were normalized by z-scores prior to analysis to allow comparability across associations. Analyses were performed unadjusted (Model 0), adjusted for age and sex (Model 1) + physical activity, smoking, and education level (Model 2) + dietary factors (Model 3). For associations with continuous MetS as a dependent variable, which already takes into account age and sex, analyses were performed unadjusted (Model 0) and fully adjusted for smoking, physical activity, education, and dietary factors (Model 3). For associations with SCORE as a dependent variable, which already takes into account age, sex, and smoking status, analyses were performed unadjusted (Model 0) and fully adjusted for physical activity, education, and dietary factors (Model 3). Dietary factors included in the fully adjusted models were dependent on the association studied, and included fish (dairy intake and FFAs), or alcohol, vegetables, fruits, and meat (dairy intake and CMD risk factors, FFAs and CMD risk factors). No other foods or food groups (listed in Table 1) were found to be strongly correlated with dairy intake, FFAs, or with CMD risk factors, and were thus not included in the models.

**Table 1:** Characteristics of the study population (*n* = 131)^a^

Characteristic	All (*n* = 131)	Men (*n* = 86)	Women (*n* = 45)	*p*-value
Demographics				
Age, years	60 (48–65)	61 (49–66)	58 (45–63)	0.11
Education, *n* (%)				0.38
Low	11 (8)	8 (9)	3 (7)	
Intermediate	48 (37)	29 (34)	19 (43)	
High	71 (55)	49 (57)	22 (50)	
Smoking status, *n* (%)				0.33
Smoker	37 (30.8)	27 (33.8)	10 (25.0)	
Non-smoker	83 (69.2)	53 (66.3)	30 (75.0)	
Diet during past month, *n* (%)				0.05*
No	124 (95)	83 (97)	41 (91)	
Yes, sometimes	4 (3)	3 (3)	1 (2)	
Yes, always	3 (2)	0 (0)	3 (7)	
Physical activity, min/week	1922 (1070–2748)	1911 (934–2725)	1930 (1215–2760)	0.44
Supplement use, *n* (%)	59 (45)	38 (44)	21 (47)	0.48
Dietary factors				
Total energy intake, kcal/day	2207 ± 507	2355 ± 477	1924 ± 443	< 0.001***
Macronutrients, g/day (% energy)				
Carbohydrates	233 ± 60 (42)	246 ± 59 (42)	208 ± 54 (43)	< 0.001***
Protein	79 ± 17 (14)	83 ± 17 (14)	71 ± 14 (15)	< 0.001***
Fat	89 ± 27 (36)	95 ± 26 (36)	79 ± 25 (37)	< 0.001***
Fiber, g/day	25 ± 8	26 ± 8	23 ± 6	0.01**
Sodium, mg/day	2357 ± 676	2524 ± 704	2040 ± 486	< 0.001***
Dairy fat, g/day	15 (9–24)	15 (9–24)	17 (10–23)	0.75
Dairy foods and groups, g/day				
Total dairy	306 (162–400)	289 (162–382)	329 (164–470)	0.18
High-fat dairy	40 (14–85)	38 (16–89)	46 (14–82)	0.85
Low-fat dairy	237 (90–354)	228 (73–316)	255 (125–381)	0.75
Total FD	151 (60–255)	152 (73–250)	151 (48–260)	0.61
High-fat FD	18 (3–49)	17 (2–49)	25 (3–49)	0.38
Low-fat FD	106 (25–226)	112 (29–220)	85 (21–230)	0.17
Total NFD	88 (30–201)	86 (26–178)	88 (37–262)	0.17
High-fat NFD	14 (6–31)	17 (6–34)	11 (5–24)	0.86
Low-fat NFD	56 (0–152)	46 (0–139)	64 (12–211)	0.09
Milk	76 (13–189)	71 (13–153)	84 (16–236)	0.36
Cheese	30 (12–50)	28 (13–52)	31 (11–46)	0.93
Yoghurt	84 (11–139)	95 (13–139)	73 (7–139)	0.84
Other foods and groups, g/day				
Coffee	406 (261–638)	406 (406–638)	406 (174–609)	0.002**
Tea	174 (65–406)	174 (27–406)	174 (67–406)	0.25
Alcoholic drinks	112 (13–289)	180 (60–386)	17 (0–112)	< 0.001***
Soft drinks	13 (0–53)	18 (0–54)	4 (0–27)	0.08
Fruits	161 (79–235)	159 (76–234)	210 (81–237)	0.60
Vegetables	139 (94–193)	129 (91–169)	160 (98–208)	0.02*
Bread	130 (96–166)	133 (101–169)	108 (76–139)	0.01*
Other cereals and grains	47 (32–88)	49 (33–100)	45 (30–84)	0.38
Potatoes	67 (37–87)	67 (37–87)	37 (22–67)	0.005**
Legumes	38 (19–75)	38 (22–78)	34 (17–73)	0.55
Meat products	74 (49–96)	84 (58–121)	66 (42–82)	0.002**
Eggs and egg products	14 (7–18)	14 (7–18)	18 (7–18)	0.62
Fish	11 (6–16)	11 (7–16)	11 (4–16)	0.88
Nuts and seeds	15 (6–26)	14 (6–26)	15 (4–27)	0.91
Sauces, spreads and cooking fats	43 (29–57)	46 (33–59)	35 (17–47)	0.001***
Salty and processed snack foods	38 (19–65)	43 (25–74)	29 (13–50)	0.01**
Sugary confectionary and desserts	78 (50–112)	79 (50–115)	68 (51–107)	0.54
Cardiometabolic factors				
BMI, kg/m^2^	25.5 (23.2–27.1)	25.8 (23.4–28.2)	24.5 (22.7–26.5)	0.06
BMI category, *n* (%)				0.10
Underweight (< 18.5 kg/m^2^)	2 (2)	1 (1)	1 (2)	
Normal weight (18.5–24.9 kg/m^2^)	58 (44)	32 (37)	26 (58)	
Overweight or obese (≥ 25–29.9 kg/m^2^)	71 (54)	53 (62)	18 (40)	
Waist circumference, cm	91.7 ± 11.6	95.0 ± 10.3	85.4 ± 11.5	< 0.001***
Diastolic blood pressure, mm Hg	74.0 ± 10.4	76.1 ± 9.7	70.0 ± 10.7	0.002**
Systolic blood pressure, mm Hg	127.9 ± 16.8	131.7 ± 15.8	120.7 ± 16.4	< 0.001***
Hypertension, *n* (%)				0.03*
Hypertension^b^	33 (25)	26 (30)	7 (16)	
Normal or optimal	98 (75)	60 (70)	38 (84)	
Hypertension treatment, *n* (%)				0.23
Being treated (with medication and/or diet)	20 (15)	16 (19)	4 (9)	
Not being treated	111 (85)	70 (81)	41 (91)	
Plasma total cholesterol, mmol/L	5.3 ± 1.0	5.3 ± 1.0	5.4 ± 1.1	0.054
Plasma LDL cholesterol, mmol/L	3.3 ± 0.9	3.4 ± 0.9	3.2 ± 0.9	0.19
Plasma HDL cholesterol, mmol/L	1.5 ± 0.4	1.4 ± 0.3	1.8 ± 0.5	< 0.001***
Serum triglycerides, mmol/L	1.0 (0.8–1.4)	1.1 (0.8–1.4)	1.0 (0.8–1.2)	0.15
Suboptimal cholesterol, n (%)	100 (76)	71 (83)	29 (64)	0.04*
High cholesterol treatment, n (%)				0.14
Being treated (with medication and/or diet)	10 (8)	9 (11)	1 (2)	
Not being treated	121 (92)	77 (89)	44 (98)	
HbA1c, mmol/mol	36.0 (34.0–37.8)	35.5 (34.0–37.0)	36.0 (34.0–38.9)	0.47
Fasting plasma glucose, mmol/L	5.3 (5.0–5.6)	5.4 (5.1–5.7)	5.0 (4.8–5.5)	< 0.001***
Diabetes, *n* (%)	2 (2)	1 (1)	1 (2)	0.38
Diabetes treatment, *n* (%)				0.40
Being treated (with medication and/or diet)	2 (1)	1 (1)	1 (2)	
Not being treated	129 (99)	85 (99)	44 (98)	
Metabolic syndrome, *n* (%)	14 (10.7)	10 (11.6)	4 (8.9)	0.74
SCORE, *n* (%)	120	80	40	0.46
≥ 15	3 (2)	3 (4)	0 (0)	
10–14	8 (7)	8 (10)	0 (0)	
5–9	25 (21)	21 (26)	4 (10)	
1–4	52 (43)	33 (41)	19 (47)	
< 1	32 (27)	15 (19)	17 (43)	

*BMI* body mass index; *FD* fermented dairy; *HDL* high-density lipoprotein; *LDL* low-density lipoprotein; *NFD* non-fermented dairy; *SCORE* Systematic COronary Risk Evaluation

^a^Values are presented as mean ± SD, or median (IQR). Missing values: education (*n* = 1), smoking status (*n* = 11), physical activity (minutes/week) (*n* = 20), LDL (*n* = 1), HDL (*n* = 1), Hb1Ac (*n* = 1), glucose (*n* = 1), SCORE (*n* = 11). Differences in characteristics between sexes were assessed using the *t*-test (for normally distributed continuous variables), Wilcoxon test (for skewed continuous variables), or chi-squared test (for categorical variables)

^b^Inclusive of Grade 1 hypertension, Grade 2 hypertension, and isolated systolic hypertension

Significant results are bolded: *p ≤ 0.05, **p ≤ 0.01, ***p ≤ 0.001

Further, we intended to examine potential mediation of the association between dairy intake and CMD risk factors by milk-derived FFAs by independently adding the FFAs to fully adjusted regression models. However, since there were no clear associations between dairy intake and CMD risk factors, we did not further examine the role of the FFAs as potential mediators.

All analyses were performed in R (Version 3.6.3) [50]. Visualizations of the intercorrelations between FFAs was performed using the corrplot R package [51], and visualizations of the associations were performed using the ggplot2 R package [52]. The script for the circular plots were adapted from Ladroue [53]. For all models, the level of significance was set at *p* ≤ 0.05. However, due to the large number of associations examined in this work, the models were also adjusted for multiple comparisons [48]. Both significant raw and FDR-adjusted *p*-values are relevant and presented; significant FDR-adjusted results are highlighted where appropriate to help focus the findings.

## Results

### Population characteristics

The characteristics of the study population are presented in Table 1. The median age of the participants was 60 years, and a majority were highly educated (55%) and non-smokers (69%). Approximately 5% of the participants were following a diet in the month preceding study enrollment. About half (54%) of the participants were overweight or obese, 33% had hypertension, 76% had suboptimal cholesterol levels, and 2% had diabetes. The distribution of the continuous MetS score and SCORE is presented in Fig. S1. Significant differences observed between men and women for several CMD risk parameters (waist circumference, blood pressure, HDL-C, plasma glucose) reflected the patterns observed in the total NQplus population [29].

The median total dairy intake of the study participants was 306 g/day, with dairy fat accounting for approximately 15 g/day (5%) of average daily energy intake. Low-fat dairy products (comprising mostly low-fat FD) were consumed at a higher level than high-fat dairy products (median: 237 vs. 40 g/day), and FD products had a higher level of intake compared to NFD products (median: 151 vs. 88 g/day). No significant differences in dairy intakes were observed between men and women. Among other dietary factors, men had significantly higher intake of total energy and several nutrients (fat, carbohydrates, fiber, protein, sodium) and foods (alcohol, potatoes, meat, sauces, and snack foods) compared to women. Significant differences were also observed for coffee intake between sexes (due to the ranking of the data by the Wilcoxon test), but medians were comparable.

Mean plasma FFA concentrations are presented in Table S3. Seventeen of the original 67 FFAs were not detected in at least a third of participants and thus removed from the analyses (primarily short-chain FFAs that are likely to be metabolized rapidly). Out of the remaining 50 FFAs, the majority were detected in plasma at concentrations of less than 1 mg/L, while five FFAs (C16, C18:1 c9, C18:2 c9c12, C18, and C20:4 c5c8c11c14) were detected at much higher concentrations compared to all other FFAs (12.6–75.3 mg/L). An inter-correlation analysis revealed that a large number of FFAs were significantly positively correlated with each other (Fig. S2).

### Free fatty acids associated with dairy intake

Among the 50 FFAs listed in Table S3, 21 were positively correlated with one of the variables of dairy intake (raw *p* ≤ 0.05) (Table S4). After adjusting for multiple comparisons, moderate correlations for dairy intake and 5 FFAs remained significant, specifically for the intake of dairy fat (C15, C16:1 t9, C16 *iso*, C17 *iso*, and C18:2 c9t11 + u; *r*_s_ = 0.26, 0.32, 0.30, 0.35, and 0.29, respectively) and cheese (C18:2 c9t11 + u; *r*_s_ = 0.30) (all FDR *p* ≤ 0.05). Out of the summed groups, summed conjugated linoleic acids (CLA) were also significantly positively correlated with dairy fat (*r*_s_ = 0.29) and cheese intake (*r*_s_ = 0.31), and summed C15 and C17, with and without C16:1 t9, was positively associated with total FD intake (*r*_s_ = 0.26) (FDR *p* ≤ 0.05).

**Fig. 1:**
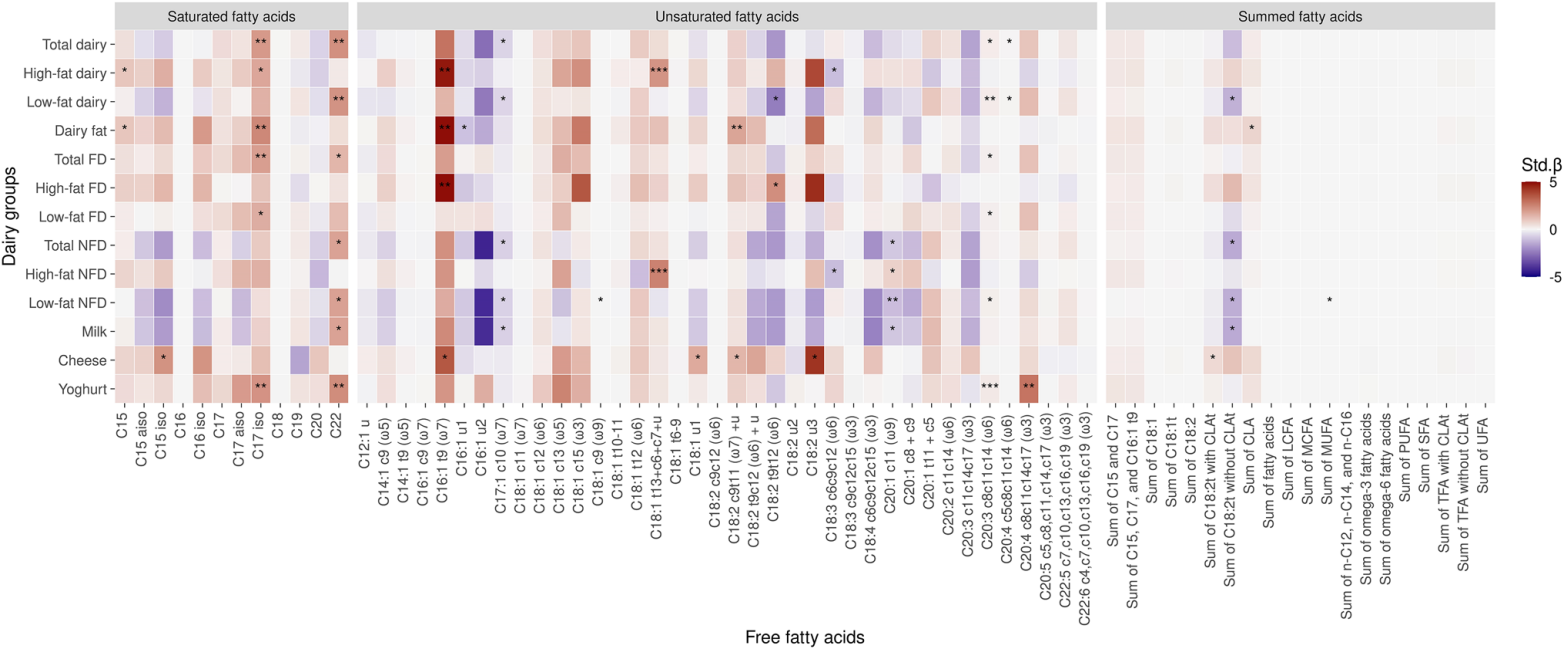
Summary of the associations between FFAs and dairy intake groups in the fully adjusted model (Model 3). Individual and summed FFAs present in more than a third of participants are included. The direction and magnitude of the standardized regression coefficient (Std. *β*) is presented as a color gradient where blue indicates positive associations and red indicates negative associations. Significance is emphasized with an asterisk: *p ≤ 0.05, **p ≤ 0.01, ***p ≤ 0.001. All significant results presented are raw *p*-values (not significant with FDR adjustment)

The correlations between FFAs and dairy intake were largely confirmed in the multiple linear regression models (Tables S5, S6). In the fully adjusted model (Fig. 1), positive associations were observed between 14 individual FFAs and multiple dairy groups/foods (raw *p* ≤ 0.05; non-significant after FDR adjustment). The strongest associations were observed between the medium-chain unsaturated FFA C16:1 t9 with dairy fat [standardized *β* (Std. *β*) = 4.9, *R*^2^ = 0.2], high-fat dairy (Std. *β* = 4.8, *R*^2^ = 0.2), high-fat FD (Std. *β* = 4.9, *R*^2^ = 0.2), and cheese (Std. *β* = 3.5, *R*^2^ = 0.1) (raw *p* ≤ 0.05). Significant associations between C15 with dairy fat (Std. *β* = 1.0, *R*^2^ = 0.1) and high-fat dairy (Std. *β* = 1.0, *R*^2^ = 0.2) intake were also observed albeit with lower effect size (no significant associations were observed for C17).

Additionally, the long-chain FFAs C17 *iso*, C22, and C20:3 c8c11c14 were found to be positively associated with the intake of multiple dairy groups/foods; the latter two FFAs were primarily associated with low-fat dairy foods/groups (raw *p* ≤ 0.05). Among the summed FFA groups, sum of CLA was positively associated with dairy fat intake, while sum of C18:2t with CLA was positively associated with cheese intake. A small number of negative associations were observed between FFAs and primarily low-fat and NFD groups (including milk), namely the long-chain unsaturated FFAs C20:1 c11, C17:1 c10, C18:2 t9t12, C18:3 c6c9c12, C18:1 c9, the medium-chain unsaturated FFA C16:1 u1, in addition to sum of C18:2t without CLA and sum of MUFA.

### Free fatty acids associated with cardiometabolic disease risk parameters

**Fig. 2:**
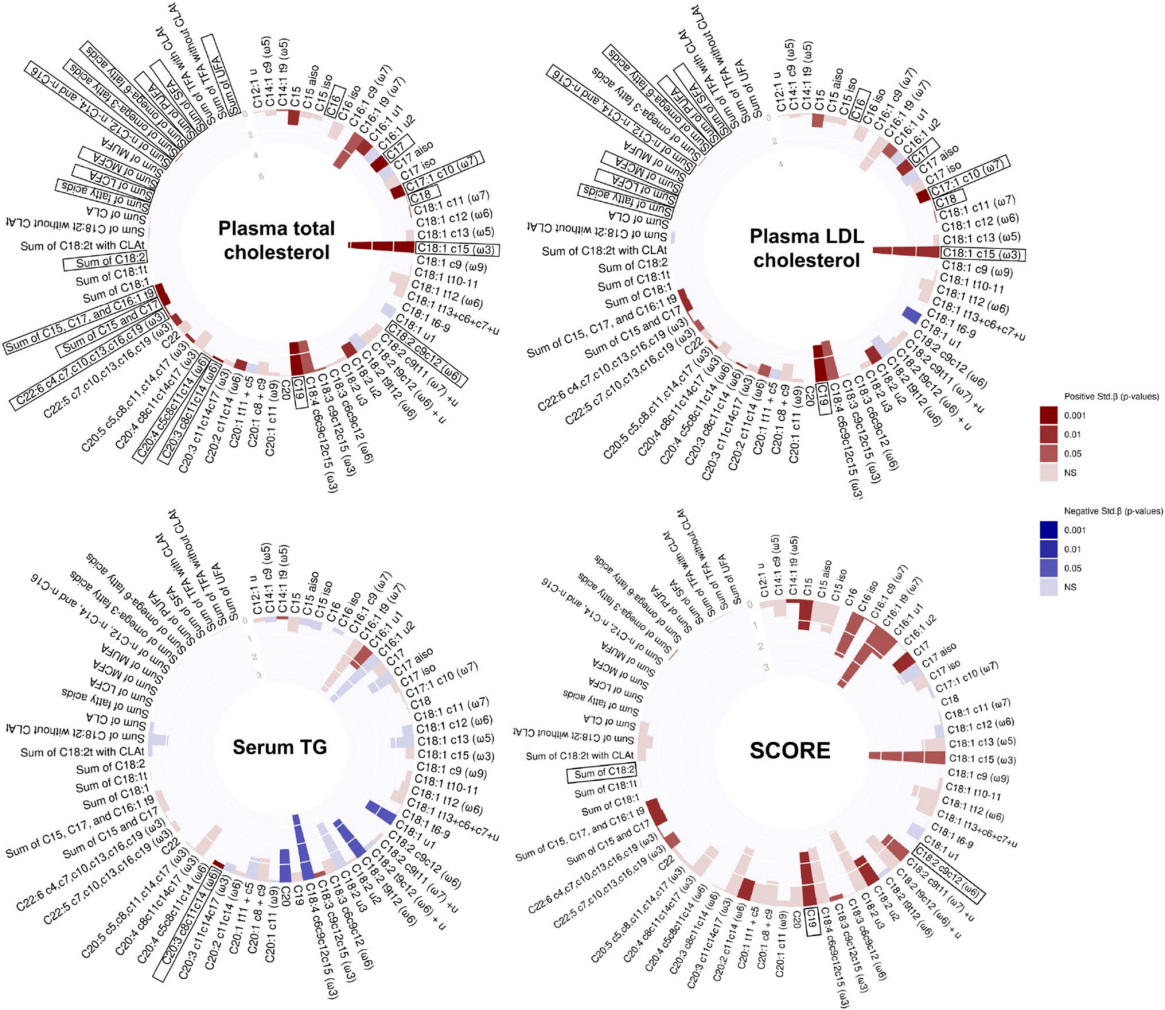
Summary of the associations between FFAs and selected CMD risk factors in the fully adjusted model (Model 3). Individual and summed FFAs present in more than a third of participants are included. The magnitude of the standardized regression coefficients (Std. *β*) are indicated in each layer of the circle plot, and the direction and significance of the associations are indicated as a color gradient. Significant FDR-adjusted associations are boxed in black. *LCFA* long-chain fatty acids; *LDL* low-density lipoprotein; *MCFA* medium-chain fatty acids; *MUFA* monounsaturated fatty acids; *NS* non-significant; *PUFA* polyunsaturated fatty acids; *SFA* saturated fatty acids; *TG* triglycerides; *UFA* unsaturated fatty acids

The results of the associations between FFAs and CMD risk parameters are presented in Tables S7 and S8. In the fully adjusted model, 33 FFAs were associated with CMD risk parameters, with the majority (30 FFAs) positively associated with increased CMD risk (raw *p* ≤ 0.05). After adjusting for multiple comparisons, associations for 10 FFAs remained significant, which involved primarily long-chain saturated (C16, C17, C18, and C19) and unsaturated FFAs (C17:1 c10, C18:1 c15, C18:2 c9c12, C20:3 c8c11c14, C20:4 c5c8c11c14, and C22:6 c4c7c10c13c16c19) (FDR *p* ≤ 0.05) (Fig. 2). These FFAs were positively associated with plasma total and LDL-C, serum triglycerides, and SCORE; the strongest associations were observed for C18:1 c15 with plasma total (Std. *β* = 6.3, *R*^2^ = 0.3) and LDL-C (Std. *β* = 5.6, *R*^2^ = 0.2) (FDR *p* ≤ 0.05). Several summed FFAs (total, medium-chain, long-chain, omega-3, omega-6, PUFA, saturated, unsaturated, as well as sum of C18:2 and sum of n-C12, n-C14, and n-C16), were also positively associated with plasma total and LDL-C, or SCORE, albeit with a low magnitude of association (Std. *β* < 0.1, FDR *p* ≤ 0.05). Sum of C15 and C17, and C15, C17, and C16:1 t9 were also positively associated with total cholesterol (Std. *β* = 0.8, FDR *p* ≤ 0.05). FFAs negatively associated with CMD risk parameters included C12:1 u with waist circumference, C20, C18:2 t9t12, C18:4 c6c9c12c15, and C18:1 u1 with serum triglycerides, and C18:1 u1 with plasma LDL-C (raw *p* ≤ 0.05; non-significant after FDR adjustment) (Fig. S3).

**Fig. 3:**
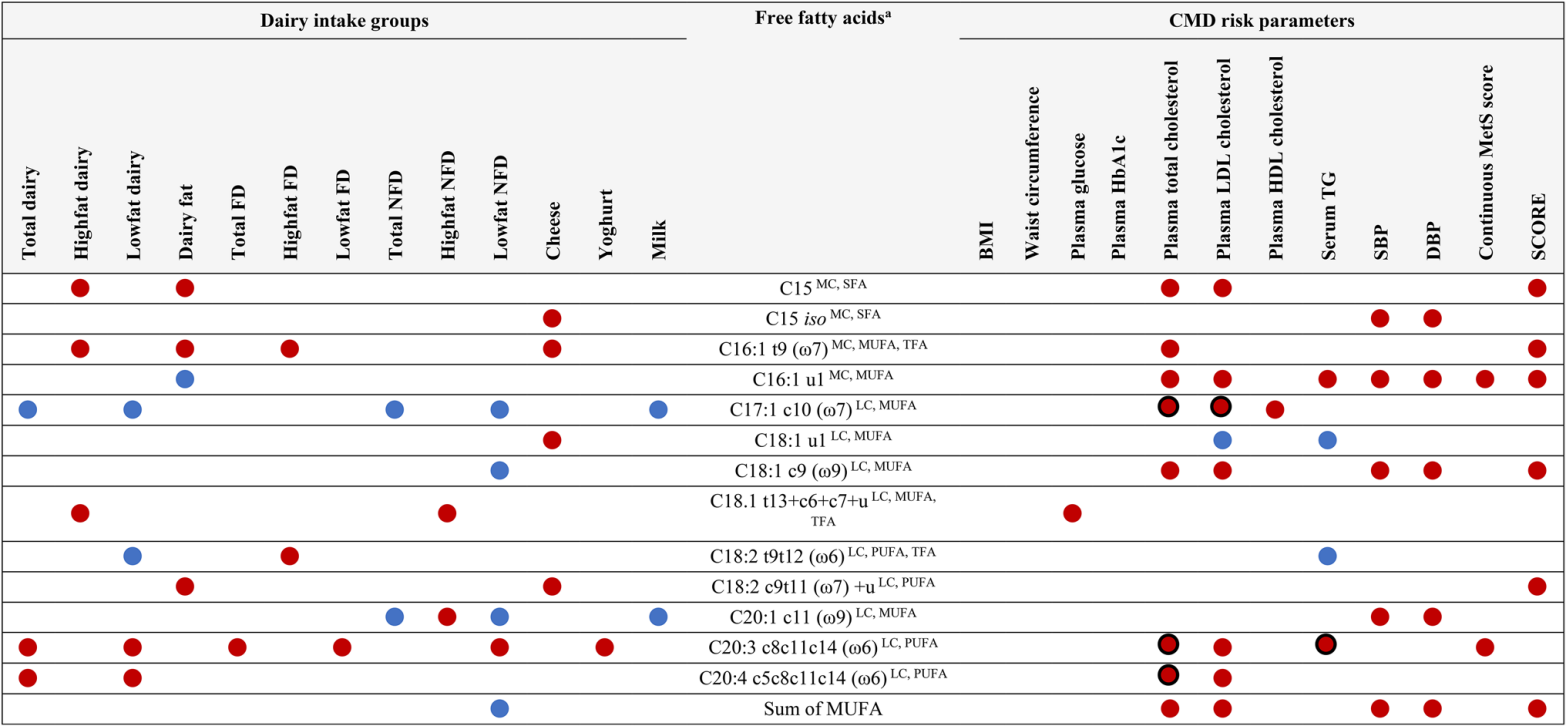
FFAs significantly associated with both dairy intake and CMD risk parameters in the fully-adjusted model (Model 3). Significant positive associations are shown with red circles, and significant negative associations are shown with blue circles, and significant FDR-adjusted associations are outlined in black (*p* ≤ 0.05). *BMI* body mass index; DBP, diastolic blood pressure; *FD* fermented dairy; *HDL* high-density lipoprotein; *LC* long-chain; *LDL* low-density lipoprotein; *MC* medium-chain; *MetS* metabolic syndrome; *MUFA* monounsaturated fatty acid; *NFD*, non-fermented dairy; *PUFA* polyunsaturated fatty acid; *SFA* saturated fatty acid; *SBP* systolic blood pressure; *SCORE* Systematic COronary Risk Evaluation

Figure 3 presents a summary of the individual and summed FFAs that were significantly associated with both dairy intake and CMD risk parameters. Of these, 5 long-chain unsaturated FFAs (C18:1 t13 + c6 + c7 + u, C18:2 c9t11 + u, C20:1 c11, C20:3 c8c11c14, and C20:4 c5c8c11c14), 2 medium-chain saturated FFAs (C15, C15 *iso*), and one medium-chain *trans* FFA (C16:1 t9) were positively associated with at least one variable of dairy intake, as well as plasma total and LDL-C, blood pressure, and SCORE. Interestingly, one long-chain PUFA, C18:2 t9t12, was positively associated with high-fat FD intake but negatively associated with serum triglyceride levels, while one long-chain saturated FFA, C18:1 u1, was positively associated with cheese intake but negatively associated with plasma LDL-C and serum triglyceride levels.

### Associations between dairy intake and cardiometabolic health

The results of the associations between dairy intake and CMD risk factors are presented in Tables S9 and S10. Only a small handful of associations were significant, but these associations attenuated in the final adjusted model (model 3), where only an association between total FD intake and low-fat FD intake and SCORE was observed (both: Std. *β* = 0.2, *R*^2^ = 0.2, raw *p* = 0.04). No associations remained significant after FDR adjustment.

## Discussion

In the current study, we performed a thorough examination of the associations between dairy intake, milk-derived FFAs, and CMD risk parameters. We found that 14 medium- and long-chain FFAs were significantly positively associated with self-reported dairy intake, particularly high-fat dairy intake and dairy fat intake. While some of these FFAs are known to be promising FIBs for dairy fat intake (e.g., C15, C16:1 t9), some of the long-chain unsaturated FFAs found to be associated with dairy intake have not been previously reported in the literature. Concurrently, significant associations were observed between 10 of these FFAs and several CMD risk parameters, particularly plasma total cholesterol and serum triglycerides. The magnitude and robustness of these relationships were maintained and were independent of adjustment for a number of demographic, lifestyle, and dietary factors. These observations suggest that these FFAs could serve a dual role as FIBs of dairy intake and in helping to inform the risk for CMD.

Several fatty acids have been previously proposed as FIBs of dairy intake, including C14, C14:1, C15, C17, C17:1, and C16:1 t9 [54, 55]. In particular, the odd-chain fatty acids C15 and C17, which are synthesized by intestinal bacteria in ruminants, have been widely used as indirect measures of dairy fat intake in association studies linking dairy intake to cardiovascular disease, stroke, and type II diabetes [14–17]. In our study, we found consistent positive associations between C15 with high-fat dairy and dairy fat intake, and C16:1 t9 with high-fat dairy, dairy fat, high-fat FD, and cheese intake (no significant associations were observed for C14, C14:1, C17, or C17:1). Significant associations observed for C18:2 c9t11 + u (a CLA found in animal fats) with dairy fat and cheese intake confirmed previous associations between this fatty acid with dairy fat intake [56]. Conversely, we also observed associations for a number of FFAs that have not previously been considered as FIBs for dairy intake, specifically the branched chain fatty acid C17 *iso* and the *trans* fatty acid C18:1 t13 + c6 + c7 + u for high-fat dairy and dairy fat intakes. A few FFAs were also found to be positively associated with low-fat dairy groups (C22) and specific dairy foods (C15 *iso*, C18:1 u1, C18:2 u3 for cheese, and C20:4 c8c11c14c17 for yoghurt). In addition, although all of the evaluated FFAs have been shown to be present in milk and should theoretically align with (self-reported) dairy intake, we found inverse associations between several FFAs and dairy fat intake, including C16:1 u1, C17:1 c10, C18:3 c6c9c12, and C20:1 c11. This finding illustrates a key conundrum in the identification and validation of FIBs, whereby metabolites found ubiquitously in multiple food sources could challenge their specificity as FIBs for dairy [57]. For instance, C17:1 is known to be a minor constituent of ruminant fats [58], yet the negative association between this fatty acid and dairy fat intake persisted regardless of adjustment for meat intake. To address this, we have recently shown that a multi-marker comprising multiple metabolites (i.e., a combination of a dairy fatty acid, a non-fatty acid dairy metabolite, and a metabolite reflecting physiological characteristics) may produce a more robust assessment of dairy food intake [30]. Nonetheless, identifying which fatty acids are strongly associated with the intake of specific dairy foods is useful for considering their inclusion in multi-marker panels that could help improve the dietary assessment of distinct dairy foods and dairy subgroups.

An important piece of insight gained in the current analysis is the combined effect of analytical method and classification of ‘high-fat’ dairy on the strength of the associations observed. Compared to our previous study where we evaluated the robustness of plasma C15 and C17 as FIBs of dairy intake study in a larger subset of the same population [30], stronger correlations were observed for the same evaluations in the current study. The improved correlations may be partly attributed to the quantitative rather than semi-quantitative approach used for the measurement of plasma free fatty acids. In particular, while we previously observed non-significant correlations between C15 with high-fat dairy and high-fat NFD groups, these correlations were significant and positive in the current study. Additionally, we observed stronger associations between several FFAs and absolute dairy fat intake, rather than high-fat dairy intake. This could be attributed to the method of classification of dairy foods as ‘high-fat’: based on what is considered a high-fat content for each dairy food (as commonly used for dairy food classification) versus based on the total fat content in the dairy food. Thus, careful consideration is needed for the classification of ‘high-fat’ dairy intake in studies where dairy biomarkers are evaluated.

Our study additionally contributes to the current state of the knowledge of using plasma FFAs as reliable biomarkers of habitual dairy fat intake. To date, there have only been a handful of reports investigating FFAs as potential FIBs of dietary fat compared with total fatty acids or fatty acids from other blood fractions, such as cholesteryl esters or serum phospholipids [59, 60]. However, these blood fractions best capture short-term dietary fat intake (past several days or hours), as opposed to long-term intake, which is best captured in erythrocyte membrane or adipose tissue [61, 62]. These biosamples can be difficult to process and store (e.g., hemolysis of erythrocytes from whole blood) or are invasive (in the case of adipose tissue) [63]. As such, they are typically not readily available in biobanks for large population-based studies. In the current report, we demonstrate that plasma FFA can be reliable FIBs of habitual dairy fat intake with associations comparable to studies measuring total FA or FA in various blood fractions.

Aside from their role as FIBs for dairy, milk-derived FFAs could play a dual role in understanding CMD risk. Of all the nutrient groups in dairy, saturated fats have arguably received the most public health attention, due to their adverse effects on circulating blood lipid profile [3, 4]. However, saturated fat is not a homogenous nutrient group, but rather consists of diverse individual fatty acids with distinct functional roles. Subgroups of medium-chain, odd-chain, and very long-chain saturated fatty acids found in dairy have been associated with lower risk of type II diabetes and improved overall metabolic health [64]. Levels of the odd-chain fatty acid C15 in plasma or serum have been linked with a higher risk of ischemic heart disease (in women) [65], but lower risk of developing type II diabetes, myocardial infarction, cardiovascular disease, and coronary heart disease [17, 66–68]. Plasma C16:1 t9 has been associated with higher LDL-C, but also with an improved metabolic profile, lower triglycerides, fasting insulin, blood pressure, and incident type II diabetes [18–20]. In our study, we observed the same heterogenous effects between individual FFAs with different indicators of CMD risk. Our analyses revealed positive associations for  ~ 30 FFAs and negative associations for 5 FFAs with different CMD risk parameters. Notably, 8 FFAs that were also significantly associated with dairy intake (including C15, C16:1 t9) were positively associated with plasma total, LDL-C, serum triglycerides, plasma glucose, blood pressure, continuous MetS, and/or SCORE, while two (C18:2 t9t12, C18:1 u1) were negatively associated with plasma LDL-C and serum triglycerides. These exploratory results suggest that distinct milk-derived FFAs could help inform different aspects of CMD risk and progression. However, causal inferences could not be established (based on the cross-sectional nature of the analyses), and thus further verification of these associations is required.

Further, we did not obtain clear associations between self-reported dairy intake and the CMD risk parameters evaluated. All of the associations were neutral and non-significant, aside from a weak positive association between total and low-fat FD intake and SCORE. The lack of significant associations could reflect the current state of the literature where, collectively, studies examining associations between dairy intake and CMD risk have produced neutral outcomes [69]. Here, the use of FIBs for dairy may better relate to the CMD outcomes by accounting for an aspect of individual variability. Evidently, examining the health impact of a single (dairy) food or food group on CMD also has its limitations, whereas examining their intake in the context of a healthy dietary pattern could offer a stronger explanation of diet-health relations. The lack of associations may also be explained by several study limitations. Our study population was small and relatively healthy, and a larger population with greater variation in CMD risk may have afforded more power to associate the dietary intake of dairy foods to CMD risk parameters. Additionally, based on the data available, we relied on a window of  ± 14 days between biosample collection and the completion of an FFQ. This assumes that dietary intakes the day prior to biosample collection were comparable to the reported intakes within the reference range of the FFQ, but otherwise, would be a source of measurement error. The use of cross-sectional data also assumes that the current diet reflects past dietary exposures that are responsible for the current disease risk and furthermore that no major dietary changes have initiated following the appearance of early disease risk markers (e.g., weight gain). Although we had a relatively healthy population, the possibility of reverse causality could not be fully excluded (e.g., awareness of elevated cholesterol prompts changes in diet for some participants). For these reasons, CMD causality could not be assessed. Notwithstanding, a strength of the current study is our comprehensive evaluation of dairy intake related biomarkers with multiple individual and composite CMD risk outcomes, which allows us to see how different FFAs associate with and impact these outcomes separately.

In conclusion, our study examining associations between dairy intake, milk-derived FFAs, and CMD risk parameters resulted in the identification of a panel of 10 medium- and long-chain FFAs that were dually associated with both dairy intake and CMD risk. The inclusion of these FFAs in future multi-marker panels could help improve the dietary assessment of different dairy foods. Further exploration in additional, larger prospective cohorts would allow the potential mediating role of these FFAs between dairy intake and CMD to be assessed, and could help confirm their role in CMD risk pathways.

## Supplementary Information

Below is the link to the electronic supplementary material.

Supplementary file 1 (PDF 1992 KB)

Supplementary file 2 (PDF 1128 KB)

Supplementary file 3 (XLSX 1730 KB)

Supplementary file 4 (XLSX 1602 KB)

Supplementary file 5 (XLSX 303 KB)

## Data Availability

All the data presented in this study can be found in the article and accompanying supplemental files.

## Code availability

All packages used in this study are cited and available from publically retrievable sources. The code used in this study is available on request from the corresponding author.
