# Supplementary material for: Associations between dairy fat intake, milk-derived free fatty acids, and cardiometabolic risk in Dutch adults
Source: Eur J Nutr. 2022 Aug 5;62(1):185–98. doi: 10.1007/s00394-022-02974-0 (PMC9899750; doi:10.1007/s00394-022-02974-0)
Supplement: Supplementary file 1 — (PDF 1992 KB) [file 394_2022_2974_MOESM1_ESM.pdf]

Supplementary Information

European Journal of Nutrition

Associations between dairy fat intake, milk-derived free fatty acids, and cardiometabolic risk in Dutch adults

Katherine J. Li[1,2]*, Elske M. Brouwer-Brolsma[1], Charlotte Fleuti[2], René Badertscher[2], Guy Vergères[2], Edith J.M. Feskens[1], Kathryn J. Burton-Pimentel[2]

[1] Division of Human Nutrition and Health, Department of Agrotechnology and Food Science, Wageningen University & Research, Wageningen, The Netherlands

[2] Agroscope, Federal Office for Agriculture (FOAG), Federal Department of Economic Affairs, Education and Research (EAER), Bern, Switzerland

* Corresponding Author

Food Microbial Systems Research Division

Agroscope, Federal Office for Agriculture (FOAG)

Federal Department of Economic Affairs, Education and Research (EAER)

Schwarzenburgstrasse 161, CH-3003 Bern

katherinejia.li@agroscope.admin.ch

Fig. S1 Distribution of cardiometabolic risk in the population, based on (a) a continuous metabolic syndrome score (age- and sex-adjusted), and (b) 10-year risk of fatal cardiovascular risk based on the European Systematic COronary Risk Evaluation (SCORE) model

(a)

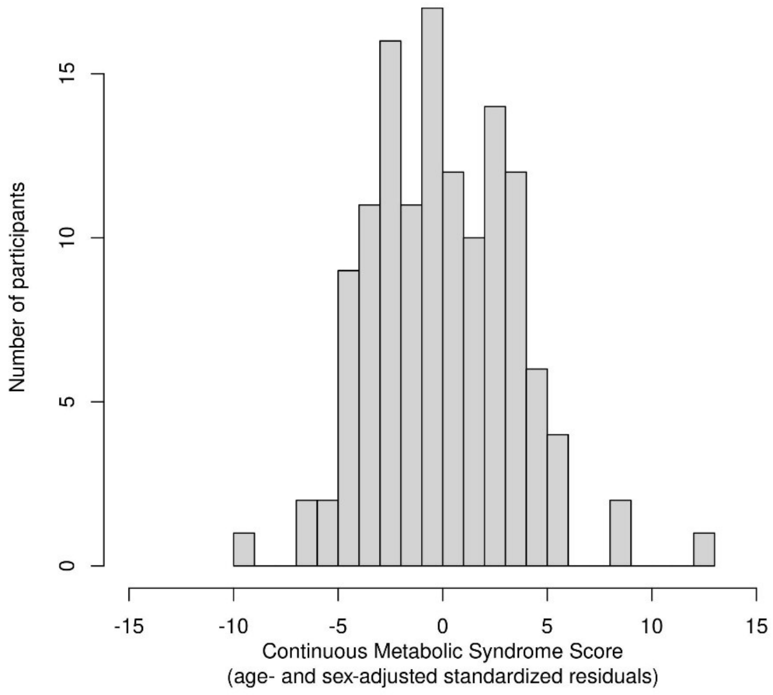

(b)

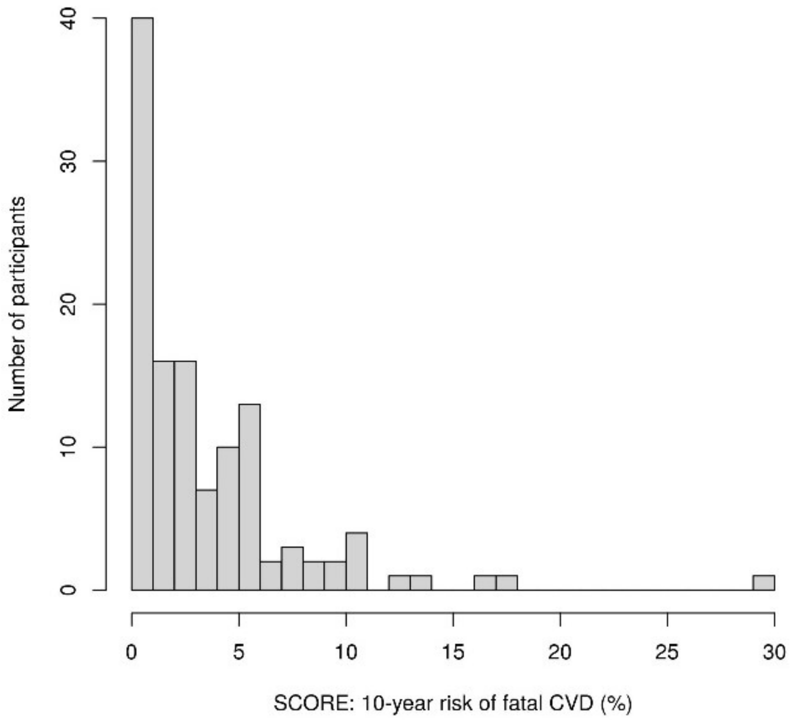

Fig. S2 Intercorrelations between free fatty acids. Individual free fatty acids present in more than a third of participants are included. The magnitude of the Spearman's correlation coefficients are represented as a colour gradient, and non-significant correlations (non-FDR adjusted p≥0.05) are indicated with an "x"

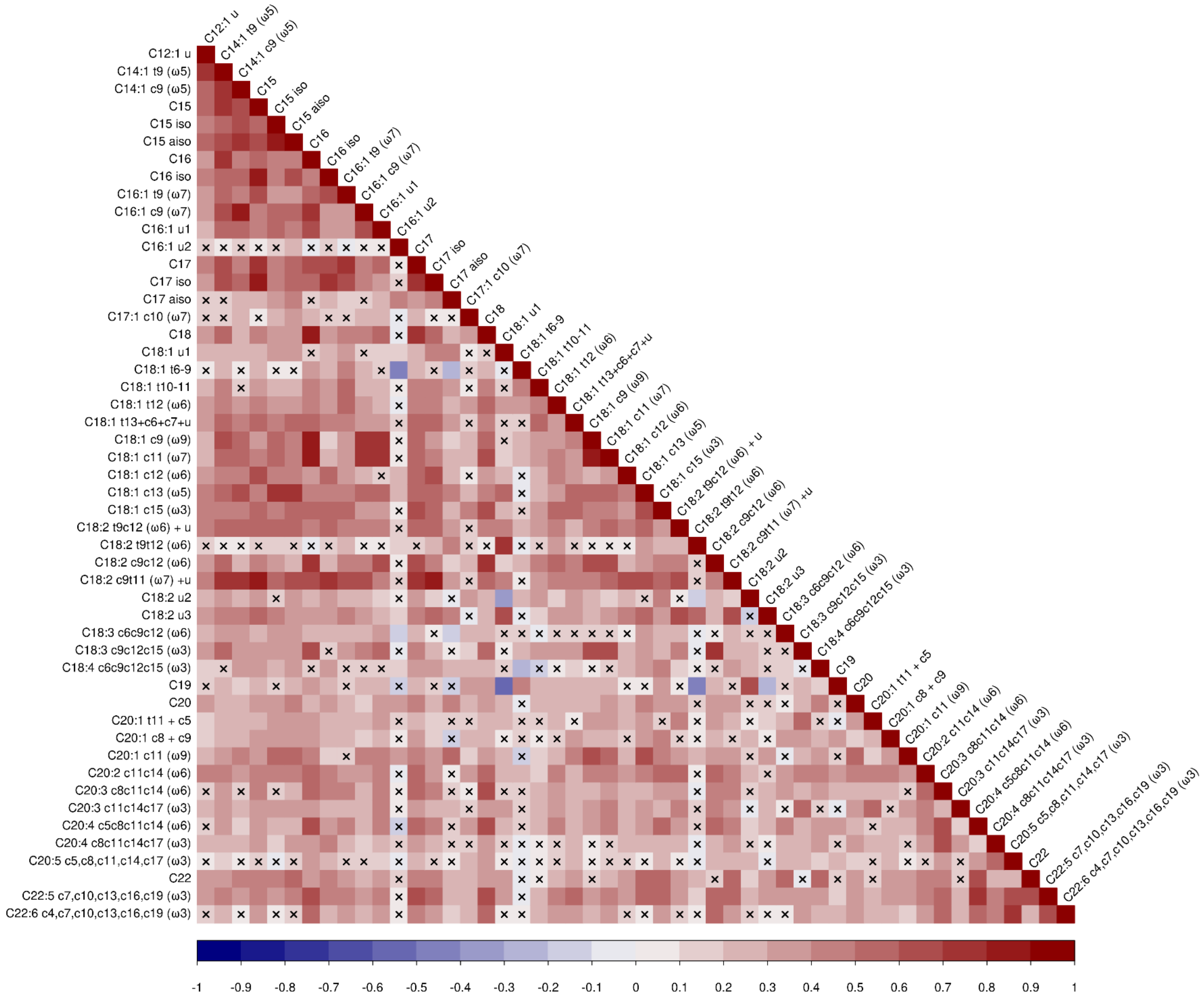

Fig. S3 Summary of the associations between free fatty acids and selected CMD risk factors in the fully-adjusted model (Model 3 – adjusted for age, sex, physical activity, education level, smoking, alcohol, vegetables, fruits, meat; for continuous MetS, adjusted for physical activity, education level, smoking, alcohol, vegetables, fruits, meat). Individual and summed free fatty acids present in more than a third of participants are included. The magnitude of the regression coefficients are indicated in each layer of the circle plot, and the direction and significance of the associations are indicated as a colour gradient. All significant results presented are raw *p*-values (not significant with FDR-adjustment). LCFA, long-chain fatty acids; LDL, low-density lipoprotein; MCFA, medium-chain fatty acids; MUFA, monounsaturated fatty acids; NS, non-significant; PUFA, polyunsaturated fatty acids; SFA, saturated fatty acids; TG, triglycerides; UFA, unsaturated fatty acids

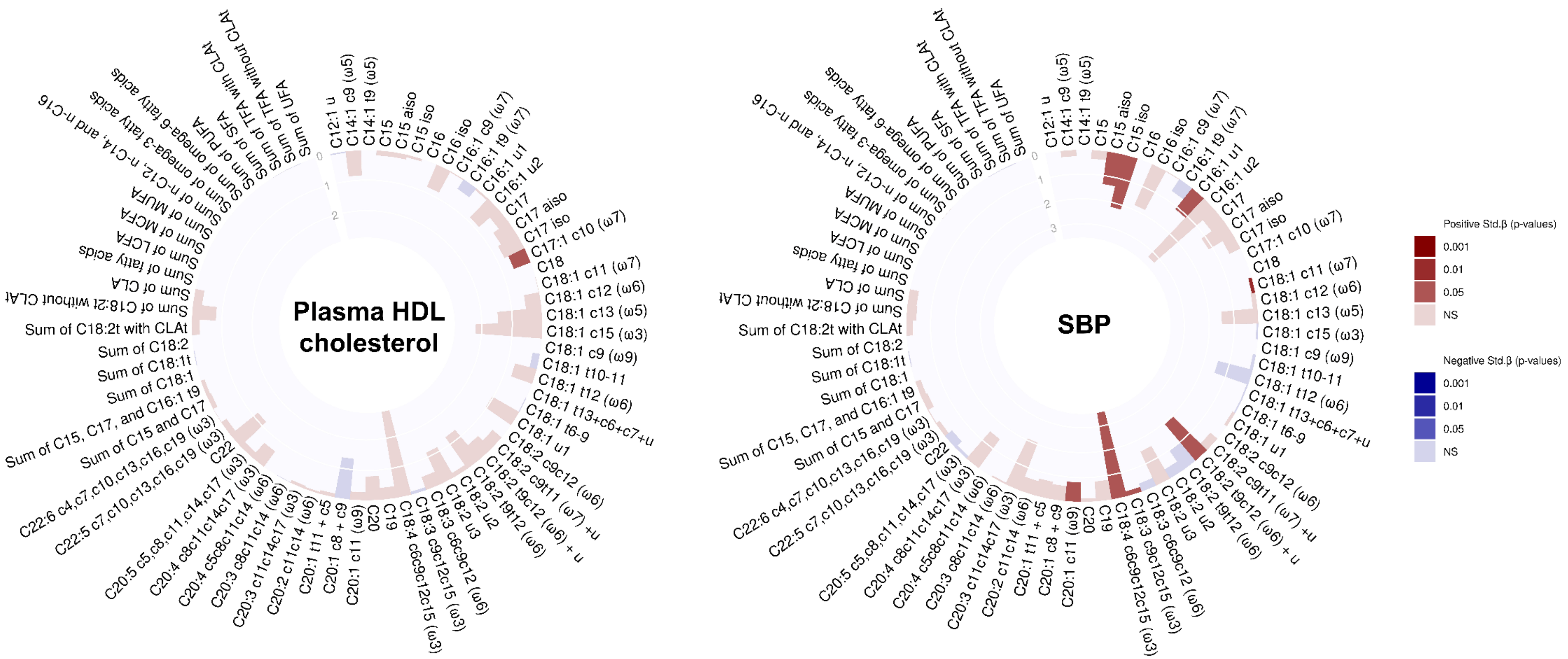

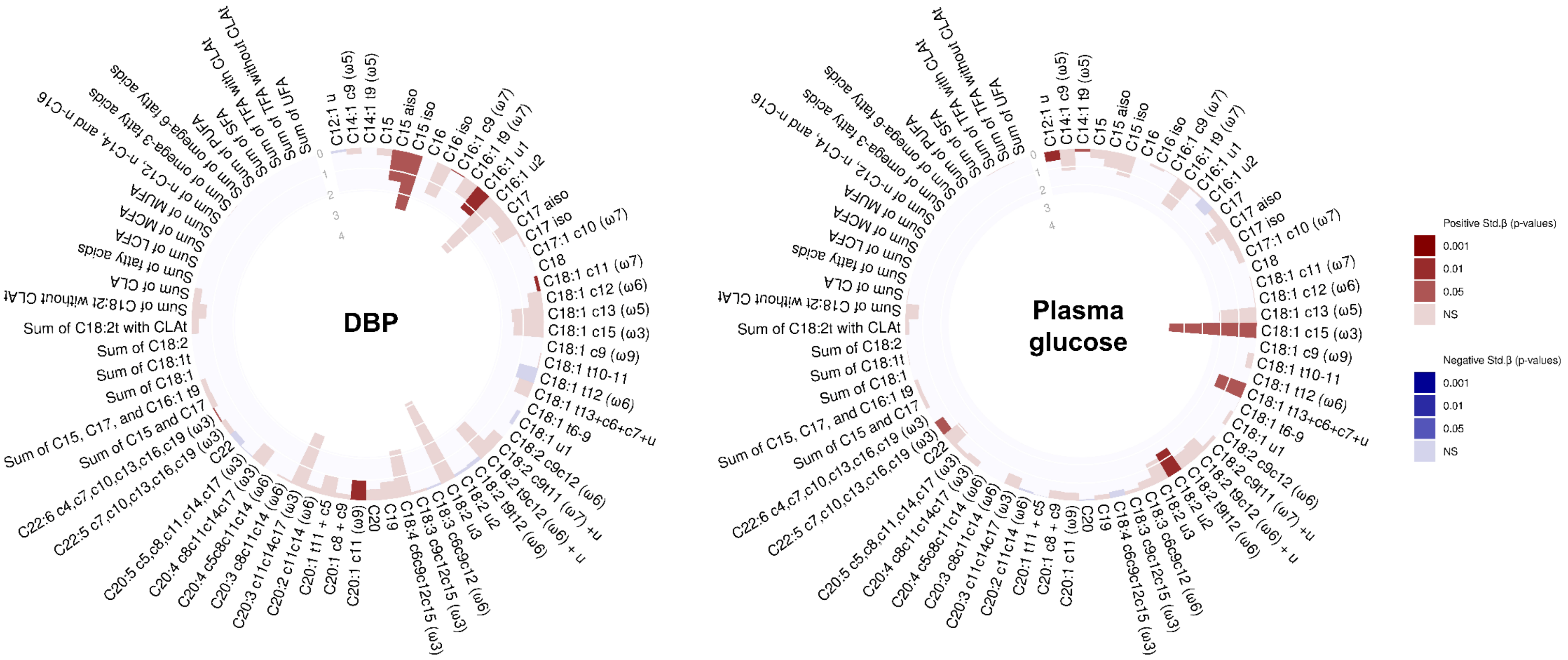
DBP
Plasma glucose
0
1
2
3
4
C12:1 u
C14:1 c9 (ω5)
C14:1 t9 (ω5)
C15
C15 aiso
C15 iso
C16
C16 iso
C16:1 c9 (ω7)
C16:1 t9 (ω7)
C16:1 u1
C16:1 u2
C17
C17 aiso
C17 iso
C17:1 c10 (ω7)
C18
C18:1 c11 (ω7)
C18:1 c12 (ω6)
C18:1 c13 (ω5)
C18:1 c15 (ω3)
C18:1 c9 (ω9)
C18:1 t10-11
C18:1 t12 (ω6)
C18:1 t13+c6+c7+u
C18:1 t6-9
C18:1 u1
C18:2 c9c12 (ω6)
C18:2 c9t11 (ω7) +u
C18:2 t9c12 (ω6) + u
C18:2 t9t12 (ω6)
C18:2 u2
C18:2 u3
C18:3 c6c9c12 (ω6)
C18:3 c9c12c15 (ω3)
C18:4 c6c9c12c15 (ω3)
C19
C20
C20:1 c11 (ω9)
C20:1 c8 + c9
C20:1 t11 + c5
C20:2 c11c14 (ω6)
C20:3 c11c14c17 (ω3)
C20:3 c8c11c14 (ω6)
C20:4 c5c8c11c14 (ω6)
C20:4 c8c11c14c17 (ω3)
C20:5 c5,c8,c11,c14,c17 (ω3)
C22
C22:5 c7,c10,c13,c16,c19 (ω3)
C22:6 c4,c7,c10,c13,c16,c19 (ω3)
Sum of C15 and C17
Sum of C15, C17, and C16:1 t9
Sum of C18:1
Sum of C18:1t
Sum of C18:2
Sum of C18:2t with CLAt
Sum of C18:2t without CLAt
Sum of CLA
Sum of fatty acids
Sum of LCFA
Sum of MCFA
Sum of MUFA
Sum of n-C12, n-C14, and n-C16
Sum of omega-3 fatty acids
Sum of omega-6 fatty acids
Sum of PUFA
Sum of SFA
Sum of TFA with CLAt
Sum of TFA without CLAt
Sum of UFA
Positive Std.β (p-values)
0.001
0.01
0.05
NS
Negative Std.β (p-values)
0.001
0.01
0.05
NS

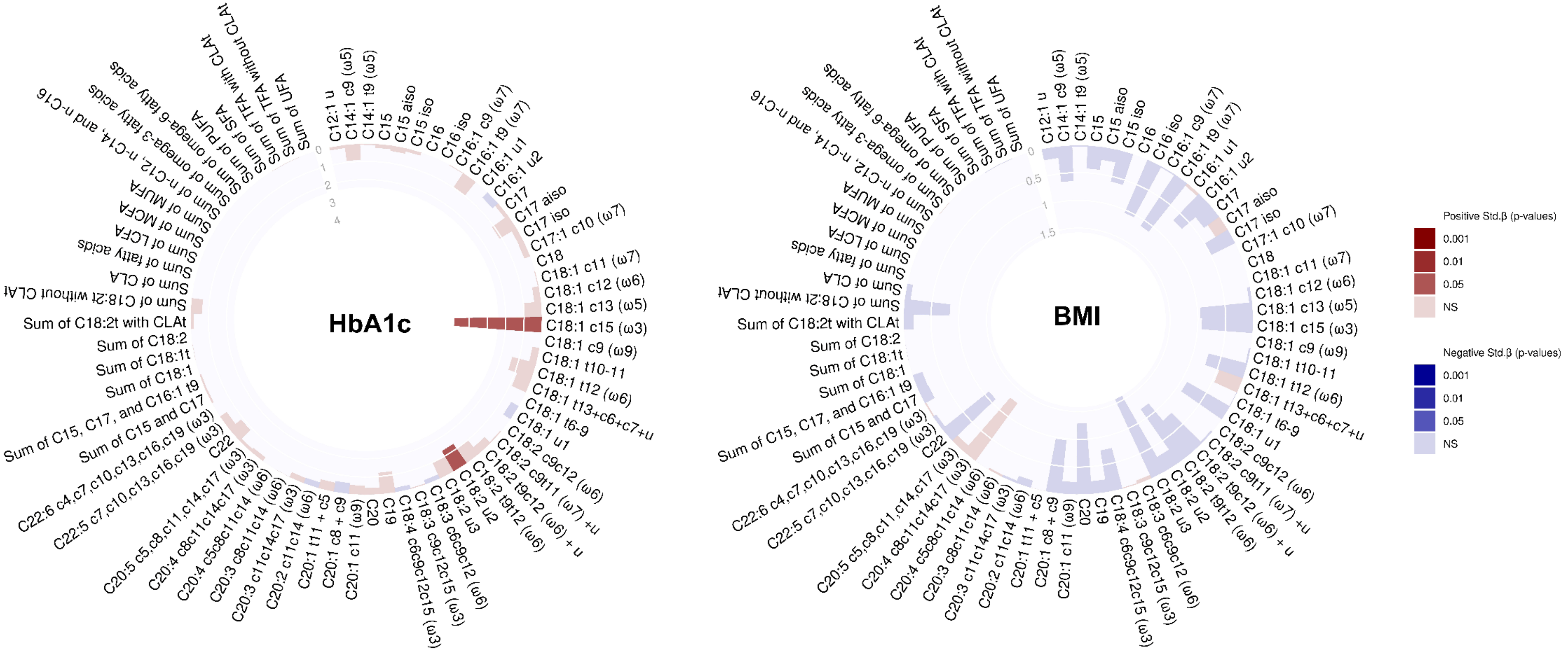

HbA1c
BMI
C12:1 u
C14:1 c9 (ω5)
C14:1 t9 (ω5)
C15
C15 aiso
C15 iso
C16
C16 iso
C16:1 c9 (ω7)
C16:1 t9 (ω7)
C16:1 u1
C16:1 u2
C17
C17 aiso
C17 iso
C17:1 c10 (ω7)
C18
C18:1 c11 (ω7)
C18:1 c12 (ω6)
C18:1 c13 (ω5)
C18:1 c15 (ω3)
C18:1 c9 (ω9)
C18:1 t10-11
C18:1 t12 (ω6)
C18:1 t13+c6+c7+u
C18:1 t6-9
C18:1 u1
C18:2 c9c12 (ω6)
C18:2 c9t11 (ω7) +u
C18:2 t9c12 (ω6) + u
C18:2 t9t12 (ω6)
C18:2 u2
C18:2 u3
C18:3 c6c9c12 (ω6)
C18:3 c9c12c15 (ω3)
C18:4 c6c9c12c15 (ω3)
C19
C20
C20:1 c11 (ω9)
C20:1 c8 + c9
C20:1 t11 + c5
C20:2 c11c14 (ω6)
C20:3 c11c14c17 (ω3)
C20:3 c8c11c14 (ω6)
C20:4 c5c8c11c14 (ω6)
C20:4 c8c11c14c17 (ω3)
C20:5 c5,c8,c11,c14,c17 (ω3)
C22:5 c7,c10,c13,c16,c19 (ω3)
C22:6 c4,c7,c10,c13,c16,c19 (ω3)
C22
Sum of C15 and C17
Sum of C15, C17, and C16:1 t9
Sum of C18:1
Sum of C18:1t
Sum of C18:2
Sum of C18:2t with CLAt
Sum of C18:2t without CLAt
Sum of CLA
Sum of fatty acids
Sum of LCFA
Sum of MCFA
Sum of MUFA
Sum of n-C12, n-C14, and n-C16
Sum of omega-3 fatty acids
Sum of omega-6 fatty acids
Sum of PUFA
Sum of SFA
Sum of TFA with CLAt
Sum of TFA without CLAt
Sum of UFA
Positive Std.β (p-values)
0.001
0.01
0.05
NS
Negative Std.β (p-values)
0.001
0.01
0.05
NS

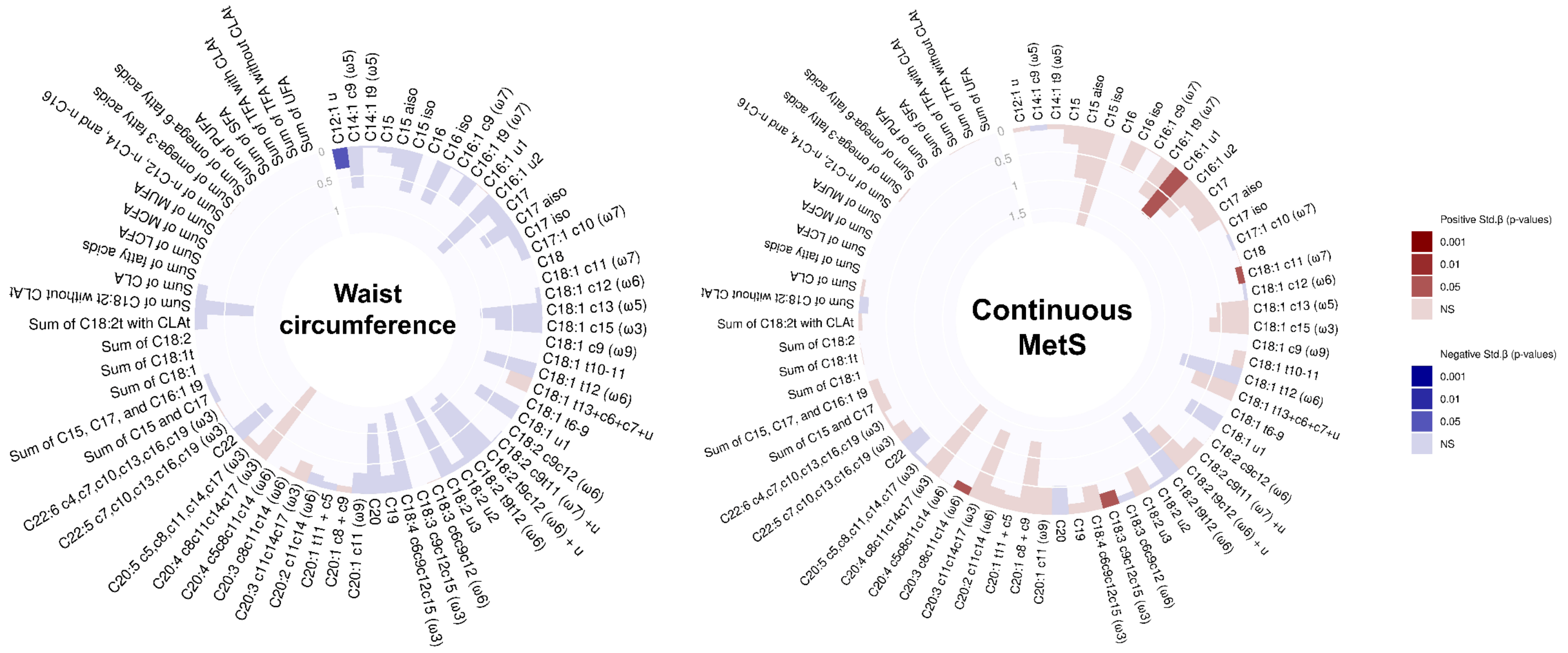

Waist circumference
Continuous MetS
0
0.5
1
1.5
C12:1 u
C14:1 c9 (ω5)
C14:1 t9 (ω5)
C15
C15 aiso
C15 iso
C16
C16 iso
C16:1 c9 (ω7)
C16:1 t9 (ω7)
C16:1 u1
C16:1 u2
C17
C17 aiso
C17 iso
C17:1 c10 (ω7)
C18
C18:1 c11 (ω7)
C18:1 c12 (ω6)
C18:1 c13 (ω5)
C18:1 c15 (ω3)
C18:1 c9 (ω9)
C18:1 t10-11
C18:1 t12 (ω6)
C18:1 t13+c6+c7+u
C18:1 t6-9
C18:1 u1
C18:2 c9c12 (ω6)
C18:2 c9t11 (ω7) +u
C18:2 t9c12 (ω6) + u
C18:2 t9t12 (ω6)
C18:2 u2
C18:2 u3
C18:3 c6c9c12 (ω6)
C18:3 c9c12c15 (ω3)
C18:4 c6c9c12c15 (ω3)
C19
C20
C20:1 c11 (ω9)
C20:1 c8 + c9
C20:1 t11 + c5
C20:2 c11c14 (ω6)
C20:3 c11c14c17 (ω3)
C20:3 c8c11c14 (ω6)
C20:4 c5c8c11c14 (ω6)
C20:4 c8c11c14c17 (ω3)
C20:5 c5,c8,c11,c14,c17 (ω3)
C22
C22:5 c7,c10,c13,c16,c19 (ω3)
C22:6 c4,c7,c10,c13,c16,c19 (ω3)
Sum of C15 and C17
Sum of C15, C17, and C16:1 t9
Sum of C18:1
Sum of C18:1t
Sum of C18:2
Sum of C18:2t with CLAt
Sum of C18:2t without CLAt
Sum of CLA
Sum of fatty acids
Sum of LCFA
Sum of MCFA
Sum of MUFA
Sum of n-C12, n-C14, and n-C16
Sum of omega-3 fatty acids
Sum of omega-6 fatty acids
Sum of PUFA
Sum of SFA
Sum of TFA with CLAt
Sum of TFA without CLAt
Sum of UFA
Positive Std.β (p-values)
0.001
0.01
0.05
NS
Negative Std.β (p-values)
0.001
0.01
0.05
NS
