# Supplementary material for: Associations between dairy fat intake, milk-derived free fatty acids, and cardiometabolic risk in Dutch adults
Source: Eur J Nutr. 2022 Aug 5;62(1):185–98. doi: 10.1007/s00394-022-02974-0 (PMC9899750; doi:10.1007/s00394-022-02974-0)
Supplement: Supplementary file 2 — (PDF 1128 KB) [file 394_2022_2974_MOESM2_ESM.pdf]

Supplementary Information

European Journal of Nutrition

Associations between dairy fat intake, milk-derived free fatty acids, and cardiometabolic risk in Dutch adults

Katherine J. Li[1,2]*, Elske M. Brouwer-Brolsma[1], Charlotte Fleuti[2], René Badertscher[2], Guy Vergères[2], Edith J.M. Feskens[1], Kathryn J. Burton-Pimentel[2]

[1] Division of Human Nutrition and Health, Department of Agrotechnology and Food Science, Wageningen University & Research, Wageningen, The Netherlands

[2] Agroscope, Federal Office for Agriculture (FOAG), Federal Department of Economic Affairs, Education and Research (EAER), Bern, Switzerland

* Corresponding Author

Food Microbial Systems Research Division

Agroscope, Federal Office for Agriculture (FOAG)

Federal Department of Economic Affairs, Education and Research (EAER)

Schwarzenburgstrasse 161, CH-3003 Bern

katherinejia.li@agroscope.admin.ch

**Table S1 Classification of Dairy Foods from the NQplus Food Frequency Questionnaire**				
**Dairy Food Item**	**Fermentation Status**	**Subgroup**	**Fat Content[a] (g/100g food)**	**Fat Classification[b]**
Buttermilk	Fermented	Buttermilk	0.2	Skim
Low-fat cheese (20+/30+)	Fermented	Cheese	14.1	Semi-skim
Regular cheese (40+)	Fermented	Cheese	23.9	Semi-skim
Regular cheese (48+)	Fermented	Cheese	30.3	Full fat
Cheese as snack	Fermented	Cheese	28.9	Semi-skim
Cheese with hot meal	Fermented	Cheese	28.9	Semi-skim
Fat luxury cheese	Fermented	Cheese	35.1	Full fat
Less-fat luxury cheese	Fermented	Cheese	22.0	Semi-skim
Unknown cheese	Fermented	Cheese	28.5	Semi-skim
(Fruit) quark with breakfast	Fermented	Quark	2.5	Skim
Full (fruit) yogurt	Fermented	Yogurt	2.8	Full fat
Full yogurt	Fermented	Yogurt	2.8	Full fat
Semi-skim (fruit) yogurt	Fermented	Yogurt	1.5	Semi-skim
Semi-skim yogurt	Fermented	Yogurt	1.8	Semi-skim
Skim (fruit) yogurt	Fermented	Yogurt	0.2	Skim
Skim yogurt	Fermented	Yogurt	0.2	Skim
Unknown yogurt	Fermented	Yogurt	1.8	Semi-skim
Butter	Non-fermented	Butter	81.1	Full fat
Skim Butter	Non-fermented	Butter	37.0	Semi-skim
Coffee cream	Non-fermented	Cream	9.4	Full fat
Cream with hot meal	Non-fermented	Cream	34.2	Full fat
Whipped cream	Non-fermented	Cream	14.8	Full fat
Milk-based ice cream	Non-fermented	Ice cream	12.0	Full fat
Diet coffee milk	Non-fermented	Milk	4.2	Full fat
Full chocolate milk	Non-fermented	Milk	2.8	Full fat
Full milk	Non-fermented	Milk	3.5	Full fat
Full-fat milk with breakfast	Non-fermented	Milk	3.5	Full fat
Regular full milk	Non-fermented	Milk	3.5	Full fat
Regular semi-skim milk	Non-fermented	Milk	1.5	Semi-skim
Semi-skim chocolate milk	Non-fermented	Milk	1.4	Semi-skim
Semi-skim coffee milk	Non-fermented	Milk	4.1	Full fat
Semi-skim milk	Non-fermented	Milk	1.5	Semi-skim
Semi-skim milk with breakfast	Non-fermented	Milk	1.5	Semi-skim
Skim chocolate milk	Non-fermented	Milk	0.5	Skim
Skim milk	Non-fermented	Milk	0.1	Skim
Unknown chocolate milk	Non-fermented	Milk	1.0	Semi-skim
Unknown coffee milk	Non-fermented	Milk	7.3	Full fat
Unknown milk	Non-fermented	Milk	1.4	Semi-skim
Milk powder for coffee	Non-fermented	Milk, powder	32.3	Full fat

[a] The fat content (g/100g) for all dairy products was determined based on the values reported in the Dutch Food Composition Table (https://nevo-online.rivm.nl/).

[b] Fat classification was based on the guidelines set by the Dutch Dairy Commodities Act (Overheid.nl. Warenwetbesluit Zuivel), where full-fat dairy included milk and milk products with a fat content >1.80%, cheeses with a fat content ≥50%, and curd cheese/quark and cream cheese with a fat content ≥35%, semi-skim dairy included milk and milk products with a fat content ≥1.50% to ≤1.80%, cheeses with a fat content >10% to <50%, and curd cheese/quark and cream cheese with a fat content ≥10% to ≤34%, and skim dairy included milk and milk products with a fat content ≤0.5%, cheeses with a fat content ≤10%, and curd cheese/quark and cream cheese with a fat content <10%. Additional qualifiers for determining the fat content of Dutch cheeses (based on fat content dry matter) included: full-fat cheese (45+ to 60+), semi-skim cheese (10+ to 40+), and skim cheese (≤10).

**Table S2 Summed free fatty acid groups**	
**Summed free fatty acid groups**	**Individual free fatty acids in group**
Total fatty acids	C4 + C5 + C6 + C7 + C8 + C9 + C10 + C10:1 (ω1) + C12 + C12:1 u + C13 *iso* + C14 + C14 *iso* + C14:1 t9 (ω5) + C14:1 c9 (ω5) + C15 + C15 *iso* + C15 *aiso* + C16 + C16 *iso* + C16:1 t9 (ω7) + C16:1 c9 (ω7) + C16:1 u1 + C16:1 u2 + C17 + C17 *iso* + C17 *aiso* + C17:1 c10 (ω7) + C18 + C18 *iso* + C18:1 u1 + C18:1 t6-9 + C18:1 t10-11 + C18:1 t12 (ω6) + C18:1 t13+c6+c7+u + C18:1 c9 (ω9) + C18:1 c11 (ω7) + C18:1 c12 (ω6) + C18:1 c13 (ω5) + C18:1 c15 (ω3) + C18:2 t10c12 (ω6) + C18:2 t9c12 (ω6)+u + C18:2 t9t12 (ω6) + C18:2 c9c12 (ω6) + C18:2 c9t11 (ω7)+u + C18:2 t9t11 (ω7) + C18:2 c9c11 (ω7)+u + C18:2 u1 + C18:2 u2 + C18:2 u3 + C18:3 c6c9c12 (ω6) + C18:3 c9c12c15 (ω3) + C18:4 c6c9c12c15 (ω3) + C19 + C20 + C20:1 t11 + c5 + C20:1 c8 + c9 + C20:1 c11 (ω9) + C20:2 c11c14 (ω6) + C20:3 c8c11c14 (ω6) + C20:3 c11c14c17 (ω3) + C20:4 c5c8c11c14 (ω6) + C20:4 c8c11c14c17 (ω3) + C20:5 c5,c8,c11,c14,c17 (ω3) + C22 + C22:5 c7,c10,c13,c16,c19 (ω3) + C22:6 c4,c7,c10,c13,c16,c19 (ω3)
Short-chain fatty acids	C4 + C5 + C6 + C7 + C8 + C9 + C10 + C10:1
Medium-chain fatty acids	C12 + C12:1 u + C13 *iso* + C14 + C14 *iso* + C14:1 t9 + C14:1 c9 (ω5) + C15 + C15 *iso* + C15 *aiso* + C16 + C16 *iso* + C16:1 t9 + C16:1 c9 + C16:1 u1 + C16:1 u2
Long-chain fatty acids	C17 + C17 *iso* + C17 *aiso* + C17:1 c10 (ω7) + C18 + C18 *iso* + C18:1 u1 + C18:1 t6-9 + C18:1 t10-11 + C18:1 t12 (ω6) + C18:1 t13+c6+c7+u + C18:1 c9 (ω9) + C18:1 c11 (ω7) + C18:1 c12 (ω6) + C18:1 c13 (ω5) + C18:1 c15 (ω3) + C18:2 t10c12 (ω6) + C18:2 t9c12 (ω6)+u + C18:2 t9t12 (ω6) + C18:2 c9c12 (ω6) + C18:2 c9t11 (ω7)+u + C18:2 t9t11 (ω7) + C18:2 c9c11 (ω7)+u + C18:2 u1 + C18:2 u2 + C18:2 u3 + C18:3 c6c9c12 (ω6) + C18:3 c9c12c15 (ω3) + C18:4 c6c9c12c15 (ω3) + C19 + C20 + C20:1 t11 + c5 + C20:1 c8 + c9 + C20:1 c11 (ω9) + C20:2 c11c14 (ω6) + C20:3 c8c11c14 (ω6) + C20:3 c11c14c17 (ω3) + C20:4 c5c8c11c14 (ω6) + C20:4 c8c11c14c17 (ω3) + C20:5 c5,c8,c11,c14,c17 (ω3) + C22 + C22:5 c7,c10,c13,c16,c19 (ω3) + C22:6 c4,c7,c10,c13,c16,c19 (ω3)
Saturated fatty acids	C4 + C5 + C6 + C7 + C8 + C9 + C10 + C12 + C13 *iso* + C14 *iso* + C14 + C15 *iso* + C15 *aiso* + C15 + C16 *iso* + C16 + C17 *iso* + C17 *aiso* + C17 + C18 *iso* + C18 + C19 + C20 + C22
n-C12, n-C14 & n-C16	C12 + C14 + C16
C18:1	C18:1 t6-9 + C18:1 t10-11 + C18:1 t12 + C18:1 t13+c6+c7+u + C18:1 c9 + C18:1 c11 + C18:1 c12 + C18:1 c13 + C18:1u1 + C18:1 c15
C18:2	C18:2 u1 + C18:2 t9t12 + C18:2 u2 + C18:2 u3 + C18:2 t9c12+u + C18:2 c9c12 + C18:2 c9t11+u + C18:2 t10c12 + C18:2 c9c11+u + C18:2 t9t11
Unsaturated fatty acids	C10:1 + C12:1 u1 + C12:1 u2 + C14:1 t9 + C14:1 c9 + C16:1 t9 + C16:1 u1 + C16:1 c9 + C16:1 u2 + C17:1 c10 + C18:1 t6-9 + C18:1 t10-11 + C18:1 t12 + C18:1 t13+c6+c7+u + C18:1 c9 + C18:1 c11 + C18:1 c12 + C18:1 c13 + C18:1 u1 + C18:1 c15 + C18:2 u1 + C18:2 t9t12 + C18:2 u2 + C18:2 u3 + C18:2 t9c12+ u + C18:2 c9c12 + C18:3 c6c9c12 + C20:1 t11+c5 + C20:1 c8+c9 + C20:1 c11 + C18:3 c9c12c15 + C18:2 c9t11+u + C18:2 + C18:2 c9c11+u + C18:2 t9t11 + C18:4 c6c9c12c15 + C20:2 c11c14 + C20:3 c8c11c14 + C20:3 c11c14c17 + C20:4 c5c8c11c14 + C20:4 c8c11c14c17 + C20:5 c5,c8,c11,c14,c17 + C22:5 c7,c10,c13,c16,c19 + C22:6 c4,c7,c10,c13,c16,c19
MUFA	C10:1 + C12:1u1 + C12:1 u2 + C14:1 t9 + C14:1 c9 + C16:1 t9 + C16:1 u1 + C16:1 c9 + C16:1 u2 + C17:1 c10 + C18:1 t6-9 + C18:1 t10-11 + C18:1 t12 + C18:1 t13+c6+c7+u + C18:1 c9 + C18:1 c11 + C18:1 c12 + C18:1 c13 + C18:1 u1 + C18:1 c15 + C20:1 t11+c5 + C20:1 c8+c9 + C20:1 c11
PUFA	C18:2 u1 + C18:2 t9t12 + C18:2 u2 + C18:2 u3 + C18:2 t9c12+u + C18:2 c9c12 + C18:3 c6c9c12 + C18:3 c9c12c15 + C18:2 c9t11+u + C18:2 t10c12 + C18:2 c9c11+u + C18:2 t9t11 + C18:4 c6c9c12c15 + C20:2 c11c14 + C20:3 c8c11c14 + C20:3 c11c14c17 + C20:4 c5c8c11c14 + C20:4 c8c11c14c17 + C20:5 c5,c8,c11,c14,c17 + C22:5 c7,c10,c13,c16,c19 + C22:6 c4,c7,c10,c13,c16,c19
C18:1t	C18:1 t6-9 + C18:1 t10-11 + C18:1 t12 (ω6) + C18:1 t13+c6+c7+u
C18:2t with CLA	C18:2 t9t12 + C18:2 t9c12+u + C18:2 c9t11+u + C18:2 t10c12 + C18:2 t9t11
CLA	C18:2 c9t11+u + C18:2 t9t11
C18:2t without CLA	C18:2 t9t12 + C18:2 t9c12+u
Trans fatty acids without CLA	C14:1 t9 + C16:1 t9 + C18:1 t6-9 + C18:1 t10-11 + C18:1 t12 + C18:1 t13+c6+c7+u + C18:2 t9t12 + C18:2 t9c12+u + C20:1 t11+c5
Trans fatty acids with CLA	C14:1 t9 + C16:1 t9 + C18:1 t6-9 + C18:1 t10-11 + C18:1 t12 + C18:1 t13+c6+c7+u + C18:2 t9t12 + C18:2 t9c12+u + C20:1 t11+c5 + C18:2 c9t11+u + C18:2 t10c12 + C18:2 t9t11
Omega-3 (ω-3) fatty acids	C18:1 c15 + C18:3 c9c12c15 + C18:4 c6c9c12c15 + C20:3 c11c14c17 + C20:4 c8c11c14c17 + C20:5 c5,c8,c11,c14,c17 + C22:5 c7,c10,c13,c16,c19 + C22:6 c4,c7,c10,c13,c16,c19
Omega-6 (ω-6) fatty acids	C18:1 t12 + C18:1 c12 + C18:2 t9t12 + C18:2 t9c12 + C18:2 c9c12 + C18:3 c6c9c12 + C18:2 t10c12 +C20:2 c11c14 + C20:3 c8c11c14 + C20:4 c5c8c11c14
Sum of C15 and C17	C15 + C17
Sum of C15, C17, and C16:1 t9	C15 + C17 + C16:1 t9

c, *cis*; CLA, conjugated linoleic acid; MUFA, monounsaturated fatty acid; PUFA, polyunsaturated fatty acid; t, *trans*; u, unknown.

Table S3 Mean concentrations of free fatty acids detected in plasma[a]	
**Free fatty acids**	**Concentration in plasma (mg/L)[a]**
**Individual free fatty acids**	
C12:1 u	0.8 ± 0.5
C14:1 c9 (ω5)	0.3 ± 0.2
C14:1 t9 (ω5)	3.5 ± 1.2
C15	0.7 ± 0.2
C15 *aiso*	0.3 ± 0.2
C15 *iso*	0.2 ± 0.1
C16	75.3 ± 17.7
C16 *iso*	0.2 ± 0.1
C16:1 c9 (ω7)	5.8 ± 2.4
C16:1 t9 (ω7)	0.2 ± 0.1
C16:1 u1	0.8 ± 0.2
C16:1 u2	0.1 ± 0.1
C17	1.1 ± 0.3
C17 *aiso*	0.1 ± 0.1
C17 *iso*	0.4 ± 0.1
C17:1 c10 (ω7)	1.3 ± 0.4
C18	30.8 ± 7
C18:1 c11 (ω7)	4.9 ± 1.6
C18:1 c12 (ω6)	0.3 ± 0.2
C18:1 c13 (ω5)	0.2 ± 0.1
C18:1 c15 (ω3)	0.2 ± 0.1
C18:1 c9 (ω9)	64.5 ± 19.9
C18:1 t10-11	1.3 ± 0.4
C18:1 t12 (ω6)	0.4 ± 0.1
C18:1 t13+c6+c7+u	0.6 ± 0.2
C18:1 t6-9	3 ± 1.4
C18:1 u1	0.2 ± 0.2
C18:2 c9c12 (ω6)	46.2 ± 11.5
C18:2 c9t11 (ω7) +u	0.4 ± 0.2
C18:2 t9c12 (ω6) + u	0.2 ± 0.1
C18:2 t9t12 (ω6)	0.2 ± 0.1
C18:2 u2	0.6 ± 0.2
C18:2 u3	0.1 ± 0.1
C18:3 c6c9c12 (ω6)	0.5 ± 0.2
C18:3 c9c12c15 (ω3)	2.2 ± 0.9
C18:4 c6c9c12c15 (ω3)	0.1 ± 0.1
C19	0.3 ± 0.1
C20	0.2 ± 0.1
C20:1 c11 (ω9)	0.7 ± 0.3
C20:1 c8 + c9	0.3 ± 0.1
C20:1 t11 + c5	0.1 ± 0.1
C20:2 c11c14 (ω6)	0.6 ± 0.3
C20:3 c11c14c17 (ω3)	0.1 ± 0.1
C20:3 c8c11c14 (ω6)	3.5 ± 1.3
C20:4 c5c8c11c14 (ω6)	12.6 ± 4.7
C20:4 c8c11c14c17 (ω3)	0.2 ± 0.1

C20:5 c5,c8,c11,c14,c17 (EPA) (ω3)	1.7 ± 1.0
C22	0.3 ± 0.1
C22:5 c7,c10,c13,c16,c19 (DPA) (ω3)	1.2 ± 0.4
C22:6 c4,c7,c10,c13,c16,c19 (DHA) (ω3)	5.6 ± 2.5
**Summed free fatty acids**	
Sum of fatty acids	296.3 ± 65.2
Sum of medium-chain fatty acids	88.2 ± 21.0
Sum of long-chain fatty acids	187.6 ± 43.3
Sum of saturated fatty acids	114.3 ± 25.5
Sum of n-C12, n-C14 & n-C16	79.6 ± 18.7
Sum of C18:1	75.5 ± 22.1
Sum of C18:2	47.9 ± 11.7
Sum of unsaturated fatty acids	161.6 ± 39.1
Sum of MUFA	85.1 ± 24.5
Sum of PUFA	76.4 ± 18.6
Sum of C18:1t	5.3 ± 1.8
Sum of C18:2t with CLA	0.8 ± 0.4
Sum of CLA	0.5 ± 0.3
Sum of C18:2t without CLA	0.4 ± 0.2
Sum of *trans* fatty acids without CLA	6.0 ± 1.9
Sum of *trans* fatty acids with CLA	6.4 ± 1.9
Sum of omega-3 fatty acids	11.5 ± 4.2
Sum of omega-6 fatty acids	64.5 ± 15.6
Sum of C15 and C17	1.8 ± 0.5
Sum of C15, C17, and C16:1 t9	2.0 ± 0.5

c, *cis*; CLA, conjugated linoleic acid; MUFA, monounsaturated fatty acid; PUFA, polyunsaturated fatty acid; t, *trans*; u, unknown.
[a] Values presented are mean ± standard deviation. Free fatty acids included in the analyses include those detected in at least one third of participants. Seventeen free fatty acids were not detected, and included: C4, C5, C6, C7, C8, C9, C10, C10:1 (ω1), C12, C13 *iso*, C14, C14 *iso*, C18 *iso*, C18:2 c9c11 (ω7) +u, C18:2 t10c12 (ω6), C18:2 t9t11 (ω7), C18:2 u1, sum of short-chain fatty acids.

Table S4 Spearman's correlations between free fatty acids and dairy intakes [a]

Free fatty acid	Spearman's correlation coefficients (r_s) – Mean energy-adjusted intakes (g/day)												
	Total dairy	High-fat dairy	Low-fat dairy	Dairy fat	Total FD	High-fat FD	Low-fat FD	Total NFD	High-fat NFD	Low-fat NFD	Cheese	Yoghurt	Milk
**Individual free fatty acids**													
C12:1 u	-0.044	0.070	-0.033	-0.062	-0.002	0.010	0.019	-0.061	0.141	-0.106	0.007	0.021	-0.052
C14:1 t9 (ω5)	-0.047	0.064	-0.054	0.047	0.000	0.009	-0.007	-0.021	0.095	-0.070	0.083	0.003	-0.048
C14:1 c9 (ω5)	0.139	0.144	0.087	0.172	0.231**	0.110	0.167	-0.056	0.094	-0.114	0.213*	0.163	-0.093
C15	0.248**	0.202*	0.172	**0.262****	0.271**	0.109	0.205*	0.115	0.178*	0.038	0.190*	0.216*	0.081
C15 *aiso*	0.094	0.110	0.046	0.103	0.145	0.047	0.091	-0.005	0.079	-0.073	0.093	0.154	-0.036
C15 *iso*	0.056	0.216*	-0.006	0.151	0.123	0.141	0.060	-0.011	0.119	-0.095	0.191*	0.144	-0.047
C16	0.070	-0.012	0.063	0.025	0.133	0.006	0.100	-0.044	-0.077	-0.017	0.084	0.121	-0.029
C16 *iso*	0.246**	0.172*	0.177*	**0.303*****	0.264**	0.128	0.199*	0.127	0.113	0.069	0.244**	0.220*	0.092
C16:1 c9 (ω7)	0.000	0.024	-0.014	-0.002	0.125	0.060	0.071	-0.174*	-0.060	-0.175*	0.112	0.069	-0.179*
C16:1 t9 (ω7)	0.206*	0.211*	0.119	**0.324*****	0.200*	0.179*	0.104	0.153	0.123	0.119	0.256**	0.147	0.140
C16:1 u1	-0.013	-0.056	-0.008	-0.148	0.053	-0.095	0.060	-0.097	-0.038	-0.126	-0.021	0.068	-0.099
C16:1 u2	-0.121	-0.041	-0.103	-0.134	-0.030	-0.071	-0.005	-0.134	0.039	-0.168	-0.097	0.031	-0.138
C17	0.220*	0.114	0.160	0.178*	0.229**	0.029	0.184*	0.120	0.128	0.064	0.102	0.233**	0.114
C17 *aiso*	-0.096	0.052	-0.098	-0.030	-0.060	-0.025	-0.071	-0.048	0.081	-0.106	-0.085	-0.021	-0.064
C17 *iso*	0.224*	0.250**	0.151	**0.352*****	0.239**	0.102	0.177*	0.158	0.281**	0.068	0.246**	0.183*	0.122
C17:1 c10 (ω7)	-0.140	-0.040	-0.138	-0.110	-0.068	-0.022	-0.035	-0.155	-0.105	-0.137	-0.054	-0.042	-0.152
C18	0.039	-0.020	0.042	0.008	0.049	-0.012	0.046	0.001	-0.033	0.029	0.015	0.076	0.023
C18:1 c11 (ω7)	-0.002	-0.012	-0.007	-0.104	0.081	-0.020	0.050	-0.113	-0.059	-0.120	-0.012	0.083	-0.095
C18:1 c12 (ω6)	0.126	0.125	0.087	0.146	0.126	0.012	0.126	0.127	0.158	0.066	0.118	0.181*	0.107
C18:1 c13 (ω5)	0.128	0.140	0.079	0.117	0.169	0.084	0.119	0.045	0.057	-0.005	0.165	0.199*	0.042
C18:1 c15 (ω3)	0.158	0.124	0.104	0.182*	0.153	0.093	0.102	0.107	0.013	0.090	0.120	0.142	0.102
C18:1 c9 (ω9)	-0.103	0.032	-0.128	-0.057	-0.010	0.038	-0.050	-0.169	-0.052	-0.193*	0.038	0.007	-0.169
C18:1 t10-11	-0.058	0.058	-0.098	0.096	-0.067	0.038	-0.109	0.016	0.016	-0.020	0.032	-0.084	0.014
C18:1 t12 (ω6)	0.063	0.044	0.056	0.069	0.045	0.069	0.024	0.087	-0.032	0.100	0.030	0.074	0.092
C18:1 t13+c6+c7+u	0.118	0.123	0.067	0.112	0.161	-0.013	0.127	0.045	0.160	-0.020	0.047	0.124	0.041
C18:1 t6-9	0.088	0.105	0.034	0.144	0.052	0.164	-0.017	0.070	-0.003	0.072	0.098	-0.005	0.077
C18:1 u1	0.032	0.199*	-0.019	0.205*	0.057	0.106	0.015	0.048	0.156	0.002	0.220*	0.104	0.016
C18:2 c9c12 (ω6)	-0.054	-0.083	-0.042	-0.044	-0.024	-0.093	-0.011	-0.063	-0.054	-0.048	-0.009	-0.026	-0.051
C18:2 c9t11 (ω7) +u	0.215*	0.133	0.171	**0.291*****	0.257**	0.080	0.206*	0.086	0.107	0.059	**0.295*****	0.183*	0.061
C18:2 t9c12 (ω6) + u	0.071	0.038	0.047	0.065	0.161	0.035	0.114	-0.025	-0.016	-0.057	0.093	0.172	-0.038
C18:2 t9t12 (ω6)	-0.073	0.177*	-0.111	0.088	-0.023	0.171	-0.079	-0.034	0.038	-0.056	0.062	0.076	-0.042
C18:2 u2	0.102	-0.047	0.099	0.006	0.176*	-0.010	0.176*	-0.034	-0.102	-0.018	-0.072	0.127	-0.021
C18:2 u3	0.142	0.228**	0.069	0.179*	0.167	0.178*	0.085	0.088	0.162	0.058	0.154	0.151	0.072
C18:3 c6c9c12 (ω6)	0.147	-0.094	0.178*	0.033	0.162	-0.069	0.176*	0.034	-0.101	0.086	0.119	0.128	0.029
C18:3 c9c12c15 (ω3)	-0.077	-0.033	-0.080	-0.102	-0.045	-0.041	-0.036	-0.065	-0.037	-0.076	-0.004	-0.036	-0.047
C18:4 c6c9c12c15 (ω3)	0.053	0.041	0.036	0.104	0.073	-0.035	0.092	0.056	0.060	0.003	0.096	0.121	0.019
C19	0.017	-0.107	0.021	-0.065	0.047	-0.046	0.045	-0.038	-0.100	-0.027	-0.131	-0.012	-0.017
C20	-0.187*	-0.031	-0.184*	-0.101	-0.097	-0.078	-0.091	-0.164	-0.035	-0.180*	-0.034	-0.065	-0.175*

C20:1 c11 (ω9)	-0.149	0.039	-0.184*	-0.133	0.001	-0.078	-0.011	-0.206*	0.081	**-0.298*** **	-0.077	-0.005	-0.220*
C20:1 c8 + c9	0.042	0.037	0.027	-0.018	0.120	-0.020	0.138	-0.025	0.005	-0.044	0.030	0.103	-0.014
C20:1 t11 + c5	0.027	0.011	0.024	-0.019	-0.024	-0.032	0.017	0.088	-0.017	0.038	0.071	-0.011	0.085
C20:2 c11c14 (ω6)	0.145	0.004	0.119	0.059	0.160	-0.057	0.170	0.066	-0.013	0.005	0.065	0.153	0.047
C20:3 c11c14c17 (ω3)	-0.093	-0.052	-0.072	-0.084	-0.115	-0.042	-0.076	0.012	-0.104	0.021	-0.035	0.000	0.018
C20:3 c8c11c14 (ω6)	0.225*	-0.036	0.236**	0.063	0.174*	-0.013	0.175*	0.132	-0.068	0.193*	0.067	0.200*	0.148
C20:4 c5c8c11c14 (ω6)	0.147	-0.049	0.179*	0.014	0.130	-0.098	0.135	0.129	-0.021	0.155	-0.001	0.121	0.145
C20:4 c8c11c14c17 (ω3)	0.115	-0.065	0.122	0.022	0.144	-0.075	0.161	-0.018	-0.072	0.029	0.121	0.160	-0.004
C20:5 c5,c8,c11,c14,c17 (EPA) (ω3)	-0.015	-0.208*	0.044	-0.144	0.033	-0.263**	0.083	-0.087	-0.070	-0.027	-0.050	-0.024	-0.059
C22	0.246**	0.064	0.239**	0.161	0.246**	0.029	0.226**	0.170	0.004	0.147	0.166	0.240**	0.155
C22:5 c7,c10,c13,c16,c19 (DPA) (ω3)	0.134	-0.090	0.166	-0.002	0.114	-0.181*	0.139	0.102	-0.027	0.124	0.039	0.117	0.119
C22:6 c4,c7,c10,c13,c16,c19 (DHA) (ω3)	0.002	-0.163	0.049	-0.138	0.011	-0.231**	0.052	-0.024	-0.041	0.015	-0.111	0.016	-0.006
**Summed free fatty acids**													
Sum of C15 and C17	0.242**	0.157	0.172*	0.221*	**0.258****	0.060	0.200*	0.119	0.161	0.050	0.136	0.229**	0.097
Sum of C15, C17, and C16:1 t9	0.252**	0.163	0.181*	0.242**	**0.259****	0.074	0.198*	0.136	0.156	0.074	0.155	0.227**	0.117
Sum of C18:1	-0.096	0.041	-0.123	-0.044	-0.011	0.048	-0.054	-0.155	-0.048	-0.178*	0.042	0.005	-0.154
Sum of C18:1t	0.068	0.103	0.012	0.147	0.036	0.113	-0.026	0.083	0.023	0.054	0.068	-0.017	0.086
Sum of C18:2	-0.046	-0.077	-0.037	-0.036	-0.014	-0.092	-0.002	-0.056	-0.046	-0.044	-0.003	-0.014	-0.045
Sum of C18:2t with CLA	0.094	0.133	0.050	0.221*	0.152	0.083	0.084	0.026	0.077	-0.004	0.231**	0.162	0.003
Sum of C18:2t without CLA	-0.031	0.168	-0.076	0.107	0.047	0.156	-0.020	-0.044	0.050	-0.076	0.103	0.137	-0.062
Sum of CLA	0.170	0.147	0.130	**0.293*** **	0.221*	0.073	0.164	0.069	0.132	0.029	**0.305*** **	0.173*	0.042
Sum of fatty acids	0.002	-0.011	-0.005	-0.013	0.063	-0.018	0.044	-0.072	-0.040	-0.062	0.041	0.072	-0.063
Sum of long-chain fatty acids	-0.019	-0.021	-0.023	-0.030	0.030	-0.036	0.017	-0.065	-0.039	-0.062	0.025	0.043	-0.054
Sum of medium-chain fatty acids	0.067	0.008	0.053	0.038	0.143	0.023	0.103	-0.059	-0.056	-0.041	0.099	0.127	-0.050
Sum of MUFA	-0.090	0.040	-0.115	-0.044	0.000	0.051	-0.044	-0.160	-0.051	-0.180*	0.046	0.013	-0.159
Sum of n-C12, n-C14, and n-C16	0.071	-0.003	0.058	0.030	0.138	0.015	0.102	-0.048	-0.069	-0.026	0.086	0.131	-0.034
Sum of omega-3 fatty acids	0.006	-0.172*	0.052	-0.119	0.026	-0.234**	0.065	-0.036	-0.066	0.009	-0.047	0.014	-0.013
Sum of omega-6 fatty acids	0.025	-0.066	0.038	-0.011	0.038	-0.083	0.043	0.002	-0.048	0.023	0.017	0.034	0.015
Sum of PUFA	0.015	-0.101	0.038	-0.043	0.032	-0.127	0.048	-0.004	-0.056	0.024	-0.007	0.032	0.011
Sum of saturated fatty acids	0.066	0.002	0.056	0.031	0.117	0.009	0.086	-0.029	-0.046	-0.007	0.072	0.118	-0.015
Sum of *trans* fatty acids with CLA	0.067	0.136	0.000	0.199*	0.062	0.144	-0.026	0.064	0.011	0.038	0.139	0.010	0.064
Sum of *trans* fatty acids without CLA	0.069	0.124	0.003	0.162	0.047	0.127	-0.025	0.075	0.024	0.044	0.093	0.003	0.078
Sum of unsaturated fatty acids	-0.042	-0.020	-0.045	-0.046	0.017	-0.031	0.001	-0.091	-0.048	-0.087	0.027	0.027	-0.081

CLA, conjugated linoleic acid; FD, fermented dairy; MUFA, monounsaturated fatty acid; NFD, non-fermented dairy; PUFA, polyunsaturated fatty acid.

[a] All significant results are indicated with an asterisk: *$p \leq 0.05$, **$p \leq 0.01$, ***$p \leq 0.001$. Significant results after adjustment for multiple comparisons (FDR-adjusted $p \leq 0.05$) are boxed in black.

Table S5 Summary of significant associations between free fatty acids and dairy groups

Free fatty acids	Significant in Model 0, 1, 2, and/or 3 [a]												
	Total dairy	High-fat dairy	Low-fat dairy	Dairy fat	Total FD	High-fat FD	Low-fat FD	Total NFD	High-fat NFD	Low-fat NFD	Cheese	Yoghurt	Milk
**Individual free fatty acids**													
C14:1 c9 (ω5)		0,1		0,1	0	1					0,1		
C14:1 t9 (ω5)		0,1		0,1		1			0,1		0,1		
C15 *iso*											0,1,3		
C15	0	0,1,2,3		0,1,2,3	0	0,1					0,1	0	
C16 *iso*				0,1							0,1		
C16:1 t9 (ω7)	0	0,1,2,3		0,1,2,3		0,1,2,3					0,1,2,3		
C16:1 u1				3									
C17 *iso*	0,1,2,3	0,1,3		0,1,2,3	0,1,2,3		2,3				0,1	0,1,2,3	
C17	0			0								0	
C17:1 c10 (ω7)	1,2,3		1,2,3					0,1,2,3		0,1,2,3			0,1,2,3
C18:1 c15 (ω3)				0									
C18:1 c9 (ω9)			0,1					0		0,1,3			0
C18:1 t13+c6+c7+u		0,1,2,3							0,1,2,3				
C18:1 t6-9				0,1		0,1							
C18:1 u1											0,1,2,3		
C18:2 c9t11 (ω7) +u				0,1,2,3							0,1,2,3		
C18:2 t9t12 (ω6)			1,2,3			2,3							
C18:2 u2					0								
C18:2 u3						2					2,3		
C18:3 c6c9c12 (ω6)		3							3				
C20:1 c11 (ω9)	0		0,1					0,1,2,3	0,1,2,3	0,1,2,3			0,1,2,3
C20:3 c8c11c14 (ω6)	0,1,2,3		0,1,2,3		0,2,3		0,2,3	0,1		0,1,2,3		0,1,2,3	0,1
C20:4 c5c8c11c14 (ω6)	3		2,3										
C20:4 c8c11c14c17 (ω3)												0,2,3	
C20:5 c5,c8,c11,c14,c17 (EPA) (ω3)		0,1		1		0,1							
C22	0,1,2,3		0,1,2,3		0,2,3		0	0,1,2,3		0,1,2,3		0,1,2,3	0,1,2,3
C22:6 c4,c7,c10,c13,c16,c19 (DHA) (ω3)											2		
**Summed free fatty acids**													
Sum of C18:1			0,1					0		0,1			
Sum of C18:1t		0,1		0,1		0,1							
Sum of C18:2t with CLA											0,2,3		
Sum of C18:2t without CLA			3			2		3		3			3
Sum of CLA				3									
Sum of MUFA			1					0		0,1,3			0
Sum of *trans* fatty acids with CLA		0,1		0,1		0,1					0,1		
Sum of *trans* fatty acids without CLA		0,1		0,1		0,1							
Sum of C15 and C17	0	0,1		0,1	0							0	
Sum of C15, C17, and C16:1 t9	0	0,1		0,1	0						0	0	

CLA, conjugated linoleic acid; FD, fermented dairy; MUFA, monounsaturated fatty acid; NFD, non-fermented dairy.

[a] Model 0 (unadjusted); Model 1 (age + sex); Model 2 (model 1 + physical activity + education level + smoking); Model 3 (model 2 + alcohol + vegetables + fruits + meat).

Free fatty acids presented are significant in at least one model.

Table S7 Summary of significant associations between free fatty acids and CMD risk factors

Free fatty acids	Significant in Model 0, 1, 2, and/or 3[a]											
	BMI	Waist circumference	Plasma glucose	Plasma HbA1c	Plasma total cholesterol	Plasma LDL cholesterol	Plasma HDL cholesterol	Serum TG	SBP	DBP	Continuous MetS score	SCORE
**Individual free fatty acids**												
C12:1 u	1,2	0,1,2,3	0,1,3									
C14:1 c9 (ω5)		1					0					
C14:1 t9 (ω5)			0,2,3		0,1,2,3	0		0,2,3				0,3
C15 *aiso*									0,1,2,3	0,1,2,3		0
C15 *iso*									0,2,3	0,1,2,3		
C15	1	1			0,1,2,3	0,1,2,3						0,3
C16 *iso*					0				0	0		0,3
C16			0,1		0,1,2,3	0,1,2,3		0,1,2,3	0	0,1,2	0,3	0,3
C16:1 c9 (ω7)			0	0			0,1		0	0,1,2,3		0,3
C16:1 t9 (ω7)					0,1,2,3	0,1,2						3
C16:1 u1					0,2,3	2,3		0,1,2,3	0,1,2,3	0,1,2,3	0,3	0,3
C16:1 u2								2		1		
C17 *aiso*								0				
C17 *iso*					0,1,2							
C17					0,1,2,3	0,1,2,3			0			0,3
C17:1 c10 (ω7)					0,1,2,3	0,1,2,3	0,1,2,3					
C18					0,1,2,3	0,1,2,3	0	0,1,2,3	0	0,1		0,3
C18:1 c11 (ω7)					0,2,3	2,3			0,1,2,3	0,1,2,3	3	0
C18:1 c13 (ω5)									0	0,1		0
C18:1 c15 (ω3)			0,1,2,3	0,2,3	0,1,2,3	0,1,2,3	0					0,3
C18:1 c9 (ω9)			0		2,3	3			0,1,2,3	0,1,2,3		0,3
C18:1 t12 (ω6)					0							
C18:1 t13+c6+c7+u			0,1,2,3									
C18:1 u1						2,3		1,2,3				
C18:2 c9c12 (ω6)					0,1,2,3	0,1,2,3			0,2,3	0,2,3		0,3
C18:2 c9t11 (ω7) +u					0		0					0,3
C18:2 t9c12 (ω6) + u									03	0,1		
C18:2 t9t12 (ω6)							0	0,1,2,3				
C18:2 u2			0,1,2,3	0,2,3	0,2,3	0,1,2,3						0,3
C18:2 u3										0,1		
C18:3 c9c12c15 (ω3)			0,1		0,2,3	0,2		0,1,2,3	0,1,2,3	0	0,3	0,3
C18:4 c6c9c12c15 (ω3)					0,2,3	0,3	0,2	1,2,3	0,1,2,3			0
C19					0,1,2,3	0,1,2,3			0,1	0,1		0,3
C20								2,3		0		
C20:1 c11 (ω9)									0,1,2,3	0,1,2,3		
C20:1 c8 + c9						0	0,1	0				0
C20:2 c11c14 (ω6)					0,2,3	0,2,3			0			0,3
C20:3 c11c14c17 (ω3)	0,1									0		0

C20:3 c8c11c14 (ω6)					0,1,2,3	0,1,2,3		0,1,2,3			0,3	
C20:4 c5c8c11c14 (ω6)					0,1,2,3	0,1,2,3	0	0,1,2				0
C20:4 c8c11c14c17 (ω3)	0,1				0,1	0		0,1	0,1	0		0
C20:5 c5,c8,c11,c14,c17 (EPA) (ω3)	0,1	0,1			0,1,2,3	0,1,3	0,1,2					0
C22					0		0,1					
C22:5 c7,c10,c13,c16,c19 (DPA) (ω3)			0,3	0	0,1,2,3	0,1,3	0	0	0	0,1		0,3
C22:6 c4,c7,c10,c13,c16,c19 (DHA) (ω3)					0,1,2,3	0,1,2,3	0	0		2,3		0,3
**Summed free fatty acids**												
Sum of C18:1			0,1		2,3	2,3			0,1,2,3	0,1,2,3		0,3
Sum of C18:2					0,1,2,3	0,1,2,3			0,2,3	0,2,3		0,3
Sum of C18:2t with CLA							0			0		
Sum of C18:2t without CLA		2										
Sum of CLA							0					
Sum of fatty acids			0		0,1,2,3	0,1,2,3		0,1	0,1,2,3	0,1,2,3		0,3
Sum of long-chain fatty acids			0		0,1,2,3	0,1,2,3		0	0,1,2,3	0,1,2,3		0,3
Sum of medium-chain fatty acids			0,1	0	0,1,2,3	0,1,2,3		0,1,2,3	0	0,1,2	0,3	0,3
Sum of MUFA			0,1		2,3	2,3			0,1,2,3	0,1,2,3		0,3
Sum of n-C12, n-C14, and n-C16			0,1		0,1,2,3	0,1,2,3		0,1,2,3	0	0,1,2	0,3	0,3
Sum of Omega-3 fatty acids					0,1,2,3	0,1,2,3	0	0,1	0	0		0,3
Sum of Omega-6 fatty acids					0,1,2,3	0,1,2,3		0,1,3	0,2,3	0,1,2,3		0,3
Sum of PUFA					0,1,2,3	0,1,2,3		0,1,3	0,2,3	0,1,2,3		0,3
Sum of saturated fatty acids			0,1		0,1,2,3	0,1,2,3		0,1,2,3	0	0,1,2	0,3	0,3
Sum of unsaturated fatty acids			0		0,1,2,3	0,1,2,3			0,1,2,3	0,1,2,3		0,3
Sum of C15 and C17	1				0,1,2,3	0,1,2,3						0,3
Sum of C15, C17, and C16:1 t9	1				0,1,2,3	0,1,2,3						0,3

BMI, body mass index; DBP, diastolic blood pressure; HDL, high-density lipoprotein; LDL, low-density lipoprotein; MetS, metabolic syndrome; MUFA, monounsaturated fatty acid; PUFA, polyunsaturated fatty acid; SBP, systolic blood pressure; SCORE, Systematic COronary Risk Evaluation.

[a] Model 0 (unadjusted); Model 1 (age + sex); Model 2 (model 1 + physical activity + education level + smoking); Model 3 (model 2 + alcohol + vegetables + fruits + meat). For continuous MetS score: Model 0 (unadjusted); Model 3 (smoking + physical activity + education level + alcohol + vegetables + fruits + meat). For SCORE: Model 0 (unadjusted); Model 3 (physical activity + education level + alcohol + vegetables + fruits + meat).

Table S9 Summary of significant associations between dairy groups and CMD risk factors

Dairy groups	Significant in Model 0, 1, 2, and/or 3						
	BMI[a]	Waist circumference[a]	Plasma HbA1c[a]	Plasma total cholesterol[a]	Plasma LDL cholesterol[a]	Serum TG[a]	SCORE[b]
Total FD				0	0		0,3
Low-fat FD			1	0			0,3
Dairy fat	2	2					
Cheese						1	
Yoghurt							

BMI, body mass index; DBP, diastolic blood pressure; FD, fermented dairy; HDL, high-density lipoprotein; LDL, low-density lipoprotein; MetS, metabolic syndrome; SBP, systolic blood pressure; SCORE, Systematic COronary Risk Evaluation.

[a] Model 0 (unadjusted); Model 1 (age + sex); Model 2 (model 1 + physical activity + education level + smoking); Model 3 (model 2 + alcohol + vegetables + fruits + meat).

[b] Model 0 (unadjusted); Model 3 (physical activity + education level + alcohol + vegetables + fruits + meat).
